# Respiratory‐Limbic Coupling via a Thalamic Circuit Alleviates Anxiety

**DOI:** 10.1002/advs.202517477

**Published:** 2026-03-02

**Authors:** Shangyu Bi, Xiaoyi Wang, Huichun Luo, Ziteng Yue, Tianjiao Deng, Yuhang Liu, Xinxin Chen, Jianxu Zhao, Luo Shi, Ning Ma, Lingyan Mao, Jing Ding, Jiwen Xu, Ti‐Fei Yuan, Sheng Wang, Fang Yuan

**Affiliations:** ^1^ Department of Neurobiology Hebei Medical University Shijiazhuang China; ^2^ Department of Anesthesiology, Renji Hospital, Key Laboratory of Anesthesiology (Shanghai Jiao Tong University), Ministry of Education Shanghai Jiao Tong University School of Medicine Shanghai China; ^3^ Department of Neurology, Zhongshan Hospital Fudan University Shanghai China; ^4^ Department of Neurosurgery Clinical Neuroscience Center Comprehensive Epilepsy Unit, Ruijin Hospital Luwan Branch Shanghai Jiao Tong University School of Medicine Shanghai China; ^5^ Shanghai Key Laboratory of Psychotic Disorders, Brain Health Institute, National Center for Mental Disorders, Shanghai Mental Health Center Shanghai Jiao Tong University School of Medicine and School of Psychology Shanghai China; ^6^ Hebei Key Laboratory of Brain Science and Brain‐Inspired Intelligence Hebei Medical University Shijiazhuang China; ^7^ The Key Laboratory of Neural and Vascular Biology Ministry of Education, Hebei Medical University Shijiazhuang China

**Keywords:** anxiety, central amygdala, paraventricular thalamic nucleus, preBötzinger complex, respiratory center

## Abstract

Breathing rhythms bidirectionally modulate affective states, yet the underlying neural pathways remain elusive. Here, we identified an ascending neural circuit that integrates respiratory patterning with affective state in male mice. This circuit originates from glutamatergic neurons in the preBötzinger complex (preBötC), projecting to the paraventricular thalamic nucleus (PVT) and subsequently targeting the central amygdala (CeA). We reveal that photostimulation of the preBötC→PVT circuit significantly alleviates acute restraint stress‐induced anxiety‐like phenotypes and reduces respiratory frequency variability. Conversely, inhibition of this circuit exacerbates anxiety‐like phenotypes and respiratory dysfunction. These effects are significantly abolished by inhibition or ablation of PVT neurons projecting to the CeA. Additionally, this anxiolytic effect is mediated by PVT projections that preferentially excite centrolateral amygdala neurons, thereby inhibiting centromedial amygdala output. Translating these findings, we show that volitional slow breathing reduces anxiety in healthy humans and suppresses anxiety‐related beta/high‐gamma oscillations in the amygdala of epilepsy patients. This work delineates a conserved respiratory‐limbic circuit that mechanistically explains the anxiolytic effect of controlled breathing.

## Graphical abstract

This study delineates a conserved preBötC^Glu^→PVT→CeA circuit that gates anxiety and respiration. Activation of this circuit is anxiolytic and respiratory‐stabilizing, while its inhibition has the opposite effect. Mechanistically, PVT exerts its anxiolytic action via a disinhibitory microcircuit: its inputs preferentially target CeL GABAergic neurons, which subsequently suppress CeM output. This model defines a dedicated ascending pathway for respiratory‐limbic integration.

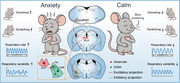

## 1 Introduction

Anxiety disorders are among the most prevalent psychiatric conditions worldwide, affecting approximately 3.3% of the global population and imposing substantial socioeconomic burdens [1]. First‐line serotonergic pharmacotherapies [2] are frequently limited by side effects, dependency risks, and variable long‐term efficacy [3]. Notably, anxiety disorders are intrinsically linked with respiratory dysfunction, as manifested by tachypnea, sleep apnea, and pathological sighing [2, 4, 5, 6], highlighting an evolutionarily conserved interplay between breathing and emotion. Consequently, breathing‐based interventions, such as deep breathing, yoga, and Tai Chi, have long been recognized as accessible, rapid‐acting strategies for mitigating stress and anxiety, demonstrating efficacy both as monotherapies and adjuvants in reducing panic and anxiety symptoms [7, 8, 9]. Despite this empirical validation, the neural circuit mechanisms underlying these behavioral benefits remain undefined, limiting the development of mechanism‐based, targeted therapies.

Breathing is a rhythmic, innate behavior governed by the respiratory central pattern generator, a highly specialized brainstem network. Within this network, the preBötzinger complex (preBötC) serves as a hub for inspiratory rhythmogenesis and pattern formation [10]. Growing evidence suggests that respiratory dynamics can significantly influence affective states [11, 12]. Forebrain regions involved in emotion and cognition processing, such as the amygdala, are modulated by respiratory signals from diverse neural sources [13]. Both clinical [14, 15] and rodent studies [16] consistently identify the amygdala, particularly its central nucleus (CeA), as a conserved node for anxiety processing [17, 18, 19, 20]. Our recent findings demonstrate that activation of the CeA→paraventricular thalamic nucleus (PVT) circuit induces anxiety‐like phenotypes in mice, while activation of the CeA→lateral parabrachial nucleus (LPBN) preferentially regulates respiratory dynamics [21]. However, while descending emotion‐to‐breathing circuits have been delineated, the ascending pathways through which respiratory rhythms gate anxiety remain uncharacterized. We therefore postulated that a dedicated brainstem→thalamus→amygdala circuit integrates breathing with affective state. The PVT emerged as a prime candidate relay, given its known connectivity with both brainstem respiratory centers and amygdala subnuclei [22, 23], as well as its established role in stress responsivity [24, 25]. Yet, its specific function in respiratory–affective integration has not been explored.

In this study, we identify and functionally characterize a glutamatergic preBötC→PVT→CeA circuit that bidirectionally gates anxiety‐like phenotypes and respiratory patterning in male mice. We demonstrate that activation of this circuit alleviates acute restraint stress (ARS)‐induced anxiety‐like phenotypes while reducing respiratory frequency (RF) and stabilizing RF variability. Conversely, circuit inhibition exacerbates both anxiety‐like phenotypes and respiratory dysregulation. Translating these preclinical insights, we further show that volitional slow breathing reduces anxiety in healthy volunteers and suppresses anxiety‐related beta/high‐gamma oscillatory activity in the amygdala of epilepsy patients. Collectively, our findings transform ancient respiratory practices from anecdotal remedies into neurobiologically grounded circuit interventions, providing a mechanistic foundation for the development of bioelectronic and behavioral therapeutics for anxiety disorders.

## 2 Results

### 2.1 Behavioral and Respiratory Changes Induced by ARS

The intricate interplay between anxiety and respiratory dynamics has been documented in prior research [26, 27]. Our recent investigations have further elucidated this relationship, revealing a robust coupling between anxiety‐like phenotypes and specific respiratory pattern alterations in mice, including prolonged grooming duration and elevated RF [21]. To probe this link in greater depth, we employed an ARS paradigm, a well‐validated model, to induce anxiety‐like phenotypes in male mice [19, 28]. Specifically, the experimental group was subjected to 30 min of complete physical confinement within restraint tubes, while the control group was exposed to their home cages containing a restraint tube for the same duration without confinement. Anxiety‐like phenotypes were subsequently evaluated using the standard open field test (OFT) and elevated plus maze (EPM) tests (Figure S1a, b). Compared to unrestrained controls, ARS‐treated mice exhibited reduced entries into and time spent in the central area of the OFT and the open arms of the EPM at 0–10 min and 30–40 min intervals post‐stress, with normalization by the 60–70 min interval (Figure S1c,d). These findings indicate that ARS‐induced anxiety‐like phenotypes are time‐dependent.

**FIGURE 1:**
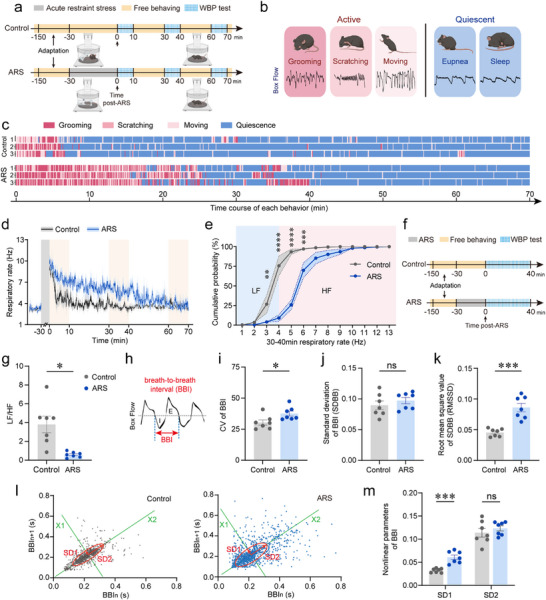
Acute restraint stress induces behavioral and respiratory dysregulation. (a) Schematic of the experimental procedure. (b) Representative respiratory waveforms corresponding to active (grooming, scratching, moving) and quiescent (eupnea, sleep) behavioral states. (c) Raster plot depicting behavioral states over a 70‑min recording period in ARS‑treated and unrestrained control mice. (d) Time‐course of RF changes. (e) Cumulative distribution of RF during the 30–40 min post‑ARS interval, with classification into low‑frequency (LF, < 4 Hz) and high‑frequency (HF, ≥ 4 Hz) breathing. (f) Schematic of the WBP procedure. (g) Ratio of LF to HF (LF/HF) calculated over a 40‑min recording session. (h) Diagram illustrating breath‐to‐breath interval (BBI). (i–k) Statistical analysis of coefficient of variation of BBI (i), standard deviation of BBI (SDBB) (j), root mean square value of SDBB (RMSSD) (k). (l) Poincaré plot of BBI with ellipse fitting (left: control; right, ARS). The coordinate system X1 and X2 is established at 45° to the normal axis. The standard deviation of the distance of the points from each axis determines the width (SD1, short‐term variability) and length (SD2, long‐term variability) of the ellipse. (m) Nonlinear parameters of BBI, derived from Poincaré analysis. Sample sizes: *n* = 3 (c), 7 (d, e, g, i–k, l, m) for each group. Statistical significance: ^*^ *p* < 0.05, ^**^ *p* < 0.01, ^***^ *p* < 0.001, ^****^ *p* < 0.0001, determined by two‐tailed unpaired *t* test or two‐tailed unpaired *t*‐test with Welch's correction (g, i–k, m), and two‐way ANOVA with Šídák's multiple comparisons tests (e). All data are presented as the mean ± SEM. Abbreviations: ARS, acute restraint stress; BBI, breath‐to‐breath interval; E, expiration; I, inspiration; RF, respiratory frequency; WBP, whole body plethysmography.

To simultaneously assess behavioral and respiratory changes, we utilized whole‐body plethysmography (WBP) in conjunction with videography, a methodology previously validated for monitoring freely behaving mice [21]. Specific breathing patterns associated with grooming and scratching serve as candidate indicators of anxiety‐like phenotypes in rodents [21, 29]. Following a 120‐min acclimatization period to the test chamber, the ARS group underwent 30 min of restraint stress, while the control group remained unrestrained. Respiratory changes were then monitored via WBP for 70 min post‐ARS (Figure 1a; Figure S1e). Based on respiratory waveforms and video recordings, behaviors were categorized into active (e.g., grooming, scratching, and moving) and quiescent (e.g., eupnea and sleep) states (Figure 1b). Representative behavior raster plots and aggregate data revealed that ARS‐treated mice exhibited prolonged active states (grooming + scratching + moving) and reduced quiescence (eupnea + sleep) during 0–10 min and 30–40 min intervals (Figure 1c; Figure S1f,g). These results align with findings from the standard OFT and EPM tests.

To comprehensively characterize respiratory dynamics, we analyzed the RF variation over a 70‐min period post‐ARS. Compared to unrestrained controls, the cumulative distribution curve of RF in the ARS‐treated mice exhibited rightward shifts during 0–10  and 30–40 min intervals, with no significant change observed during the 60–70 min interval (Figure 1d,e; Figure S1h,i), indicative of stress‐induced tachypnea bouts. Moreover, RF variability was further assessed over a 40‐min period post‐ARS (Figure 1f). Defining low‐frequency (LF) RF as <4 Hz and high‐frequency (HF) RF as ≥4 Hz, we observed a significant decrease of the LF/HF ratio in the ARS‐treated mice compared to unrestrained controls (Figure 1g). Time domain and nonlinear analyses of the breath‐to‐breath interval (BBI) were also performed (Figure 1h). Time domain analysis revealed significantly increased RF variability in ARS‐treated mice, as evidenced by heightened coefficients of variation (CV) (Figure 1i) and increased root mean square value of standard deviation (RMSSD) of BBI (Figure 1k), while the standard deviation of BBI (SDBB) remained unchanged (Figure 1j). Nonlinear analysis using Poincaré plots of BBI provided additional insights into the global structure of RF variability by mapping the relationship between consecutive respiratory cycles (Figure 1l). This analysis demonstrated a significant increase in SD1, reflecting short‐term variability, in ARS‐treated animals relative to unrestrained controls, while SD2, reflecting long‐term variability, showed no significant alteration (Figure 1m). Collectively, ARS‐induced anxiety‐like phenotypes are characterized by prolonged active behaviors (e.g., grooming and scratching), reduced quiescent time, elevated RF, and increased RF variability. These findings underscore the intricate interplay between stress‐induced respiratory dysregulation and behavioral manifestations of anxiety, extending the standard approach for assessing anxiety in animal models.

### 2.2 Anatomical Connection Linking the preBötC to the CeA via PVT Neurons

The preBötC is a key structure responsible for inspiratory rhythmogenesis [10], while the CeA serves as a primary hub for processing anxiety [30]. Given the established link between breathing rhythms and affective states [12, 21], we sought to map the neural substrates connecting these two brain regions.

**FIGURE 2:**
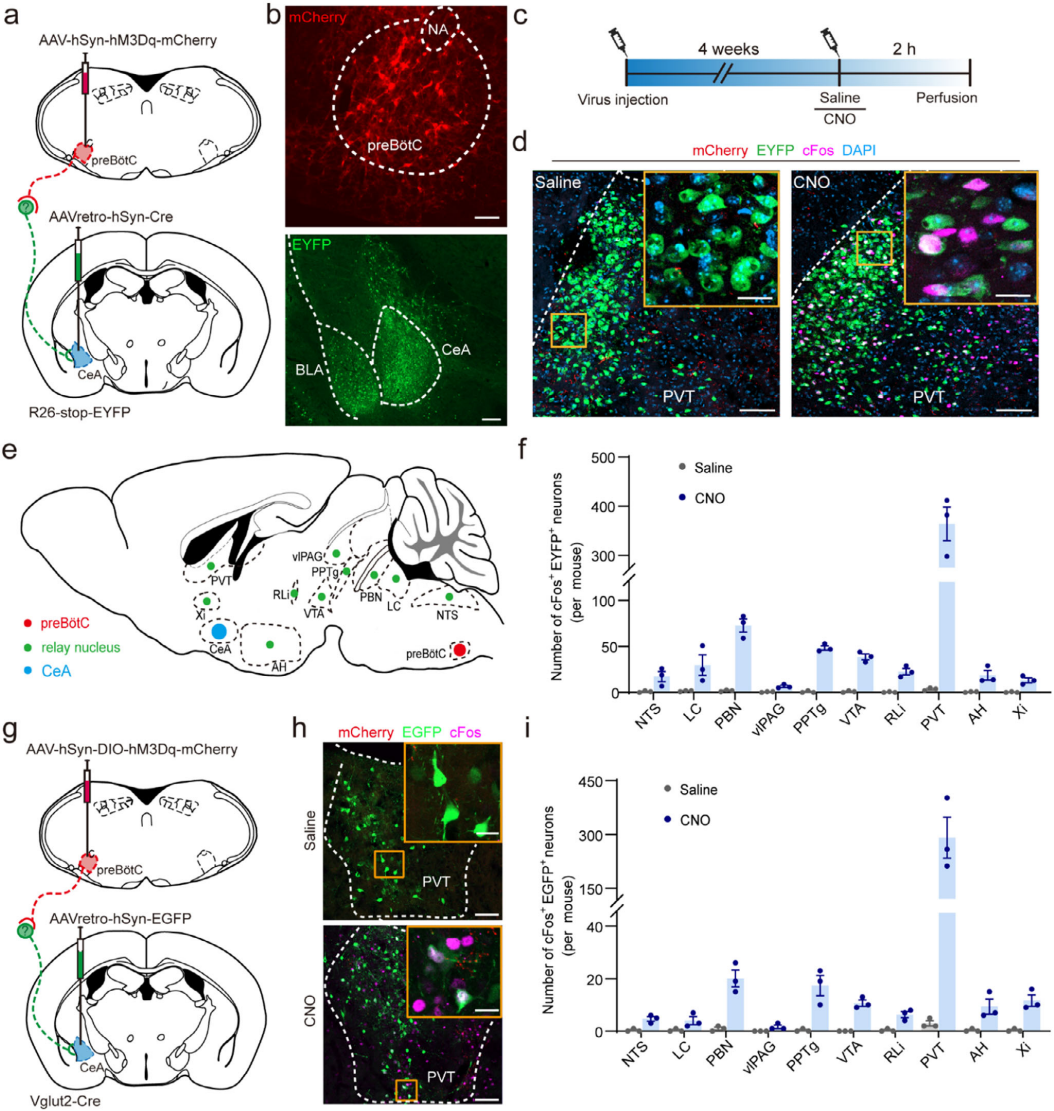
Identification of the PVT as a key relay nucleus between the preBötC and the CeA. (a) Schematic of strategies for neural tracing and chemogenetic stimulation in R26‐stop‐EYFP mice. (b) Immunohistochemical confirmation of hM3Dq‐mCherry expression in preBötC neurons and EYFP^+^ neurons in the CeA. Scale bars, 200 µm. (c) Schematic of the timeline of chemogenetic stimulation. (d) Representative images of the PVT following saline (left) or CNO (right) injection. CNO administration increased the number of EYFP^+^cFos^+^ neurons in the PVT, indicating activation of preBötC‑recruited neurons that project to the CeA. Insets show higher magnification of boxed regions. Scale bars: 100 µm (overview), 20 µm (insets). (e) Whole‑brain map of candidate relay nuclei (green) receiving input from the preBötC (red) and projecting to the CeA (blue). Regions include the brainstem (NTS), pons (LC, PBN), midbrain (vLPAG, PPTg, RLi, VTA), thalamus (PVT, Xi), and hypothalamus (AH). (f) Quantification of cFos^+^EYFP^+^ neurons across the mapped regions. (g) Schematic of the chemogenetic and tracing strategy in Vglut2‑Cre mice. (h) Images showing EGFP^+^ PVT neurons projecting to the CeA. EGFP^+^cFos^+^ neurons were significantly increased in the PVT after CNO (bottom) versus saline (top), confirming activation of preBötC^Glu^‑recruited PVT neurons that target the CeA. Scale bars: 100 µm (overview), 20 µm (insets). (i) Quantification of cFos^+^EGFP^+^ neurons in the PVT and other candidate relays. Sample sizes: *n* = 3 mice per group (f, i). Abbreviations: AH, anterior hypothalamic area; BLA, basolateral amygdaloid nucleus, anterior; CeA, central amygdala; LC, locus coeruleus; NA, nucleus ambiguous; NTS, nucleus tractus solitarii; PBN, parabrachial nucleus; PPTg, pedunculo‐pontinetegmental nucleus; PVT, paraventricular thalamic nucleus; RLi, rostral linear nucleus; vlPAG, ventrolateral periaqueductal gray; VTA, ventral tegmental area; Xi, xiphoid thalamic nucleus.

We employed a combination of chemogenetic stimulation and neural tracing approaches to examine the anatomical and functional connections between the preBötC and the CeA. The AAV encoding hM3Dq‐mCherry under the hSyn promoter (AAV‐hSyn‐hM3Dq‐mCherry) was unilaterally delivered into the preBötC of R26‐stop‐EYFP mice, while the retrograde tracing virus AAVretro‐hSyn‐Cre was injected into the ipsilateral CeA (Figure 2a). Four weeks post‐injection, immunohistochemistry confirmed the presence of mCherry^+^ preBötC neurons and EYFP^+^ axons in the CeA (Figure 2b). For chemogenetic stimulation, mice were administered clozapine‐N‐oxide (CNO; 2 mg kg^−1^, i.p.) or an equivalent volume of saline, followed by immunohistochemical assays 2 h post‐injection (Figure 2c). This approach enabled the activation of preBötC neurons and their downstream targets, including those neurons projecting to the CeA. Neurons innervated by the preBötC and those projecting to the CeA were identified based on cFos^+^, EYFP^+^, or cFos^+^EYFP^+^ labeling, with cFos^+^EYFP^+^ neurons likely representing relay neurons mediating communication between the preBötC and CeA. Notably, CNO administration significantly increased the number of cFos^+^ neurons in both the preBötC (Figure S2a,b) and CeA (Figure S2c,d) compared to saline injection, confirming the efficacy of chemogenetic stimulation. However, mCherry^+^ axon projections from the preBötC were not prominently detected in the CeA, suggesting that preBötC neurons primarily influence CeA activity through indirect synaptic pathways.

To identify relay neurons activated by preBötC neurons and concurrently projecting to the CeA, we mapped their distribution across the brainstem, thalamus, and other regions, including the nucleus tractus solitarius (NTS), locus coeruleus (LC), parabrachial nucleus (PBN) (Figure S3a–j). Notably, a substantial number of cFos^+^EYFP^+^ neurons was observed in the PVT in CNO‐treated mice compared to controls (Figure 2d), with the PVT exhibiting a significantly higher density of cFos^+^EYFP^+^ neurons relative to other regions (Figure 2e,f). These findings suggest that the preBötC may modulate behavioral states by engaging these relay neurons in the PVT, which in turn influences the CeA activity. A subset of glutamatergic neurons in the preBötC (hereafter called preBötC^Glu^ neurons) has been implicated in generating inspiratory rhythms [31]. Using a similar approach (Figure 2g), we identified relay neurons transmitting information from preBötC^Glu^ neurons to CeA neurons in Vglut2‐Cre mice. Likewise, mice received an injection of CNO (2 mg kg^−1^, i.p.) or saline, followed by immunohistochemical analysis. Quantitative assessment revealed a greater number of cFos^+^EGFP^+^ neurons in the PVT compared to other brain regions (Figure 2h,i). Given that the majority of PVT neurons are glutamatergic [32], RNAscope fluorescence in situ hybridization (RNAscope‐FISH) further confirmed that PVT neurons predominantly expressed *Slc17a6* RNA (indicative of glutamatergic neurons) rather than *Slc32a1* RNA (Figure S4a,b).

In short, we suggest that stimulation of preBötC neurons enhances activation levels of CeA neurons through relay neurons in specific brain regions, with the preBötC→PVT→CeA circuit emerging as a key candidate pathway. Subsequent investigations aimed to examine whether manipulation of this circuit contributed to the regulation of anxiety‐like phenotypes.

### 2.3 Inhibition of preBötC^Glu^ Neurons Projecting to the PVT Exacerbates Stress‐Induced Anxiety‐Like Phenotypes and Respiratory Dysregulation

**FIGURE 3:**
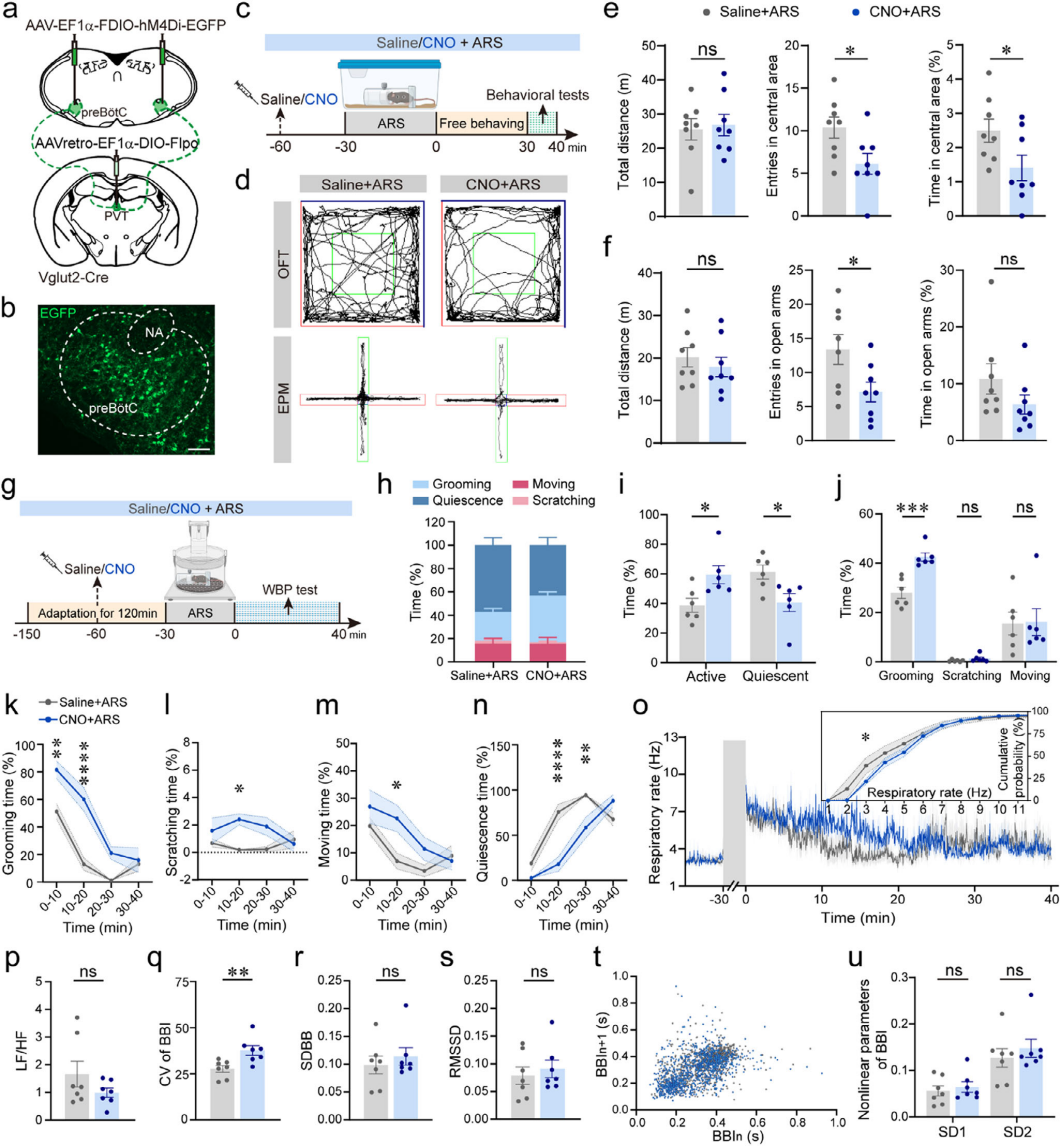
Inhibition of preBötC^Glu^‒PVT signaling exacerbates ARS‐induced anxiety‐like phenotypes and respiratory dysregulation. (a) Schematic of viral strategy for chemogenetic inhibition of the preBötC^Glu^ **‒**PVT pathway in Vglut2‑Cre mice. (b) Immunohistochemical validation of hM4Di‐EGFP expression in preBötC^Glu^ neurons projecting to the PVT. Scale bar, 100 µm. (c) Diagram of the experimental protocol of behavioral tests. (d) Representative trajectories of the OFT (top) and EPM (bottom). (e,f) Chemogenetic inhibition of the preBötC^Glu^‐PVT pathway with CNO injection (vs. saline) significantly decreased the number of entries into and time in the central area of the OFT (e) and the number of entries into open arms of EPM tests (f). (g) Schematic of the WBP procedure. (h) Percentage distribution of time spent in grooming, scratching, moving and quiescence over a 40‐min recording. (i) Cumulative percentage time in active vs. quiescent states. (j) Cumulative duration of each active behavior. (k–n) Percentage of duration of grooming (k), scratching (l), moving (m), and quiescence (n) in 10‐min bins. (o) Time‐course traces of RF. Insert: cumulative distribution plot of RF across the 40‑min session. (p) Ratio of low‑frequency to high‑frequency breathing (LF/HF). (q–s) Statistical analyses of CV (q), SDBB (r), RMSSD (s) of BBI. (t) Poincaré plot of BBI. (u) Short‑term (SD1) and long‑term (SD2) variability derived from Poincaré analysis. Sample sizes: *n* = 8 mice (e,f), = 6 (h–o), = 7 (p–s, u) for each group. Statistical significance: All data are presented as the mean ± SEM. ^*^ *p* < 0.05, ^**^ *p* < 0.01, ^***^ *p* < 0.001, ^****^ *p* < 0.0001, determined by two‐way ANOVA with Šídák's multiple comparisons tests (k–o) and two‐tailed unpaired *t*‐test (with Welch's correction or Mann‑Whitney test where appropriate; e, f, i, j, p–s, u).

To interrogate the behavioral relevance of the preBötC^Glu^→PVT→CeA circuit, we employed chemogenetic inhibition by injecting AAV‐EF1α‐fDIO‐hM4Di‐EGFP into the preBötC and AAVretro‐EF1α‐DIO‐Flpo into the PVT of Vglut2‐Cre mice (Figure 3a). Immunohistochemical imaging confirmed hM4Di‐EGFP expression in the preBötC (Figure 3b). We first assessed behavioral consequences of chemogenetic inhibition of preBötC^Glu^ neurons projecting to the PVT using the OFT and EPM tests in both ARS‐treated mice (Figure 3c) and unrestrained controls (Figure S5a). In ARS‐treated mice, administration of CNO, compared to saline, significantly reduced the number of entries and time spent in the center field of the OFT and the open arms of the EPM (Figure 3d–f). However, neither CNO nor saline injection induced significant behavioral changes in the unrestrained controls under identical experimental conditions (Figur S5b–d). Therefore, chemogenetic inhibition of preBötC^Glu^ neurons projecting to the PVT exacerbates ARS‐induced anxiety‐like phenotypes.

To further examine the effects of chemogenetic inhibition on respiratory and behavioral dynamics, we performed WBP recordings (Figure 3g; Figure S5e). Mice were acclimated to the WBP chamber for 2 h before receiving injections of either saline or CNO in both ARS‐treated and unrestrained control groups. The ARS‐treated group was then subjected to 30 min of restraint stress, while controls remained unrestrained. Respiratory changes were recorded for 40 min post‐treatment. Quantitative analysis revealed that CNO administration significantly increased active time and reduced quiescent time in ARS‐treated mice compared to saline injection (Figure 3h–j). Behavioral assessments conducted every 10 min over the 40‐min period demonstrated that chemogenetic inhibition of preBötC^Glu^ neurons projecting to the PVT led to increased grooming, scratching, and moving behaviors at different time points, alongside reduced quiescence following ARS (Figure 3k–n).

Analysis of RF variability revealed that mice receiving CNO exhibited a decreased LF/HF ratio, with the cumulative distribution map showing a reduced percentage of RF ≤ 3 Hz (Figure 3o,p), indicating an increase in high‐frequency breathing bouts. Time domain analysis revealed a significantly higher CV of BBI in CNO‐injected mice compared to saline controls (Figure 3q). However, no significant differences were observed in SDBB, RMSSD, and Poincaré plot parameters (SD1 and SD2) between the two groups (Figure 3r–u). Despite this anxiety‐exacerbating effect in ARS‐treated mice, inhibition of the preBötC^Glu^→PVT circuit did not induce significant behavioral or respiratory changes in unrestrained controls (Figure S5f–s), with the exception of increased CV of BBI in CNO‐injected mice (Figure S5o). Take together, inhibition of the preBötC^Glu^→PVT circuit exacerbates anxiety‐like phenotypes in ARS‐treated mice, as evidenced by prolonged grooming time, reduced quiescent time, elevated RF and heightened RF variability.

### 2.4 Activation of the preBötC^Glu^→PVT Circuit Alleviates ARS‐Induced Anxiety‐Like Phenotypes and Respiratory Dysregulation

**FIGURE 4:**
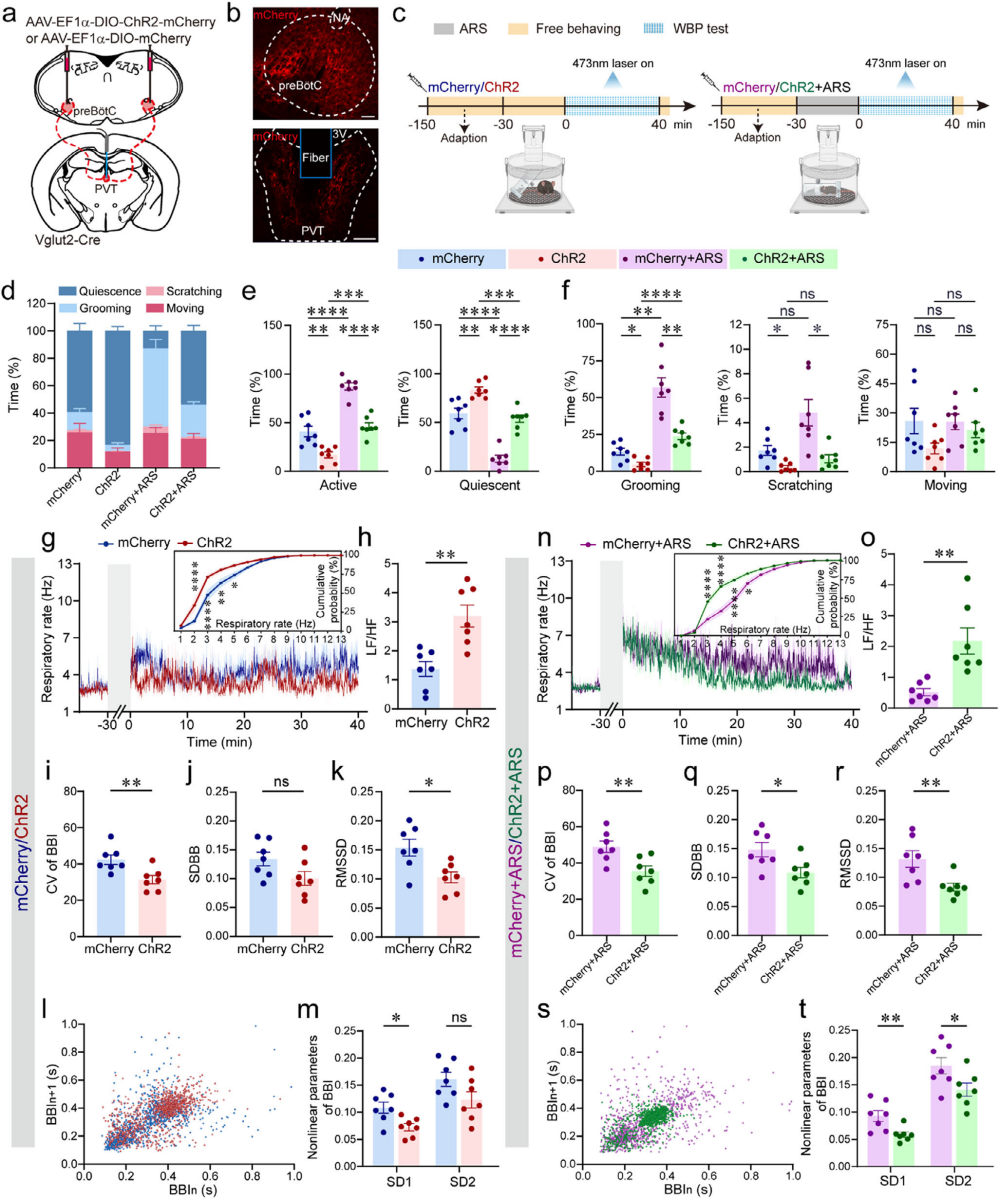
Photostimulation of axon terminals of preBötC^Glu^ neurons to the PVT alleviates ARS‐induced anxiety‐like phenotypes and respiratory dysregulation. (a) Schematic of viral strategy for optogenetic activation of preBötC^Glu^ axon terminals in the PVT of Vglut2‐Cre mice. (b) Representative images showing ChR2‐mCherry expression in preBötC somata and corresponding axonal projections within the PVT. Scale bars, 100 µm. (c) Schematic of the experimental procedure. (d) Percentage of time spent in quiescent or active states (grooming, scratching, moving) during a 40‐min recording. (e) Accumulative time spent in active vs. quiescent state. (f) Proportion of total time allocated to specific active behaviors. (g) RF over time under baseline (unrestrained) conditions. Inset: Cumulative RF distribution from mCherry‐control versus ChR2‐mCherry‐expressing mice. (h) Ratio of LF to HF (LF/HF). (i–k) Quantitative analysis of CV (i), SDBB (j) and RMSSD (k) of BBI. (l) Poincaré plot of BBI. (m) Nonlinear variability parameters derived from Poincaré analysis. (n) RF over time following ARS. Inset: Cumulative RF distribution from ARS‐treated mice expressing mCherry‐control or ChR2‐mCherry. (o) LF/HF ratio post‐ARS. (p–r) Quantitative analysis of CV (p), SDBB (q) and RMSSD (r) of BBI. (s) Poincaré plot of BBI. (t) Nonlinear variability parameters (SD1, SD2) from Poincaré analysis post‐ARS. Sample sizes: *n* = 7 (d–k, m–r, t) for each group. Statistical significance: All data are presented as the mean ± SEM. ^*^ *p* < 0.05, ^**^ *p* < 0.01, ^***^ *p* < 0.001, ^****^ *p* < 0.0001, determined by one‐way ANOVA with Bonferroni's or Tamhane's T2 multiple comparisons tests (e,f), two‐way ANOVA with Šídák's or Bonferroni's multiple comparisons tests (g,n) and two‐tailed unpaired *t*‐test (with Welch's correction or Mann‐Whitney test where appropriate; h–k, m, o–r, t). Abbreviations: 3 V, third ventricle.

To investigate the impact of activating the preBötC^Glu^→PVT circuit on anxiety‐like phenotypes and respiratory phenotypes, we employed an optogenetic stimulation strategy by injecting a viral vector encoding ChR2 (AAV‐EF1α‐DIO‐ChR2‐mCherry) into the preBötC of Vglut2‐Cre mice, while control mice received injection of a virus lacking ChR2 (AAV‐EF1α‐DIO‐mCherry) (Figure 4a). Immunohistochemistry confirmed ChR2‐mCherry expression in the preBötC and its axon terminals in the PVT (Figure 4b). We subsequently assessed the impact of photostimulation of the preBötC^Glu^→PVT circuit on anxiety‐like phenotypes using the OFT and EPM in both ARS‐treated and unrestrained mice (Figure S6a). Photostimulation (473 nm, 10 mW, 20 ms pulse width, 10 Hz, 10 min) of preBotC^Glu^ neuron axon terminals within the PVT significantly increased entries into and time spent in the center field of the OFT (Figure S6b,c) and the open arms of the EPM test (Figure S6d,e) in the ChR2‐injected animals compared to the mCherry‐injected counterparts, regardless of ARS‐treated or unrestrained mice. These findings indicate that the preBötC^Glu^→PVT circuit activation significantly alleviates anxiety‐like phenotypes.

WBP experiments were conducted to further evaluate the effect of circuit activation on behavioral and respiratory dynamics. Following acclimatization to the WBP chamber, mice were subjected to either restraint or unrestraint conditions for 30 min, after which photostimulation was applied during a 40‐min WBP recording session (Figure 4c). In both ARS and unrestrained groups, photostimulation of preBötC^Glu^ neuron axon terminals within the PVT considerably reduced active time and increased quiescent time in ChR2‐injected mice compared to mCherry‐injected counterparts (Figure 4d,e), as manifested by decreased duration of grooming and scratching behaviors (Figure 4f). Meanwhile, a leftward shift in the RF cumulative distribution curve and an elevated LF/HF ratio were observed in ChR2‐injected unrestrained mice compared to mCherry‐injected counterparts (Figure 4g,h), indicating low‐frequency breathing bouts. Concomitantly, ChR2‐injected animals exhibited significant changes in RF variability metrics, including CV, RMSSD and SD1 (Figure 4i–m). In ARS‐treated mice, photostimulation also remarkably decreased RF (Figure 4n,o) and significantly reduced the RF variability parameters including CV, SDBB, RMSSD, SD1 and SD2 (Figure 4p–t). Notably, activation of the preBötC^Glu^→PVT circuit effectively alleviates ARS‐induced anxiety‐like phenotypes and respiratory dysregulation, as evidenced by reduced active behaviors, increased quiescent behaviors, diminished RF and RF variability.

We also investigated whether photostimulation of the GABAergic preBötC neurons projecting to the PVT affects anxiety‐like phenotypes in unrestrained mice using OFT and EPM tests. We injected AAV‐EF1α‐DIO‐ChR2‐mCherry or the control virus AAV‐EF1α‐DIO‐mCherry into the preBötC of Vgat‐Cre mice. Four weeks post‐injection, immunohistochemistry validated the presence of mCherry expression in the preBötC and PVT (Figure S7a). Photostimulation caused no significant changes in behavioral parameters of OFT and EPM tests between ChR2‐injected mice relative to control mice (Figure S7b–g). Hence, preBötC^GABA^ neurons projecting to the PVT do not appear to modulate anxiety‐like phenotypes.

### 2.5 Inactivation of the PVT→CeA Projection Eliminates Anxiolytic Effects of preBötC^Glu^→PVT Circuit Activation

**FIGURE 5:**
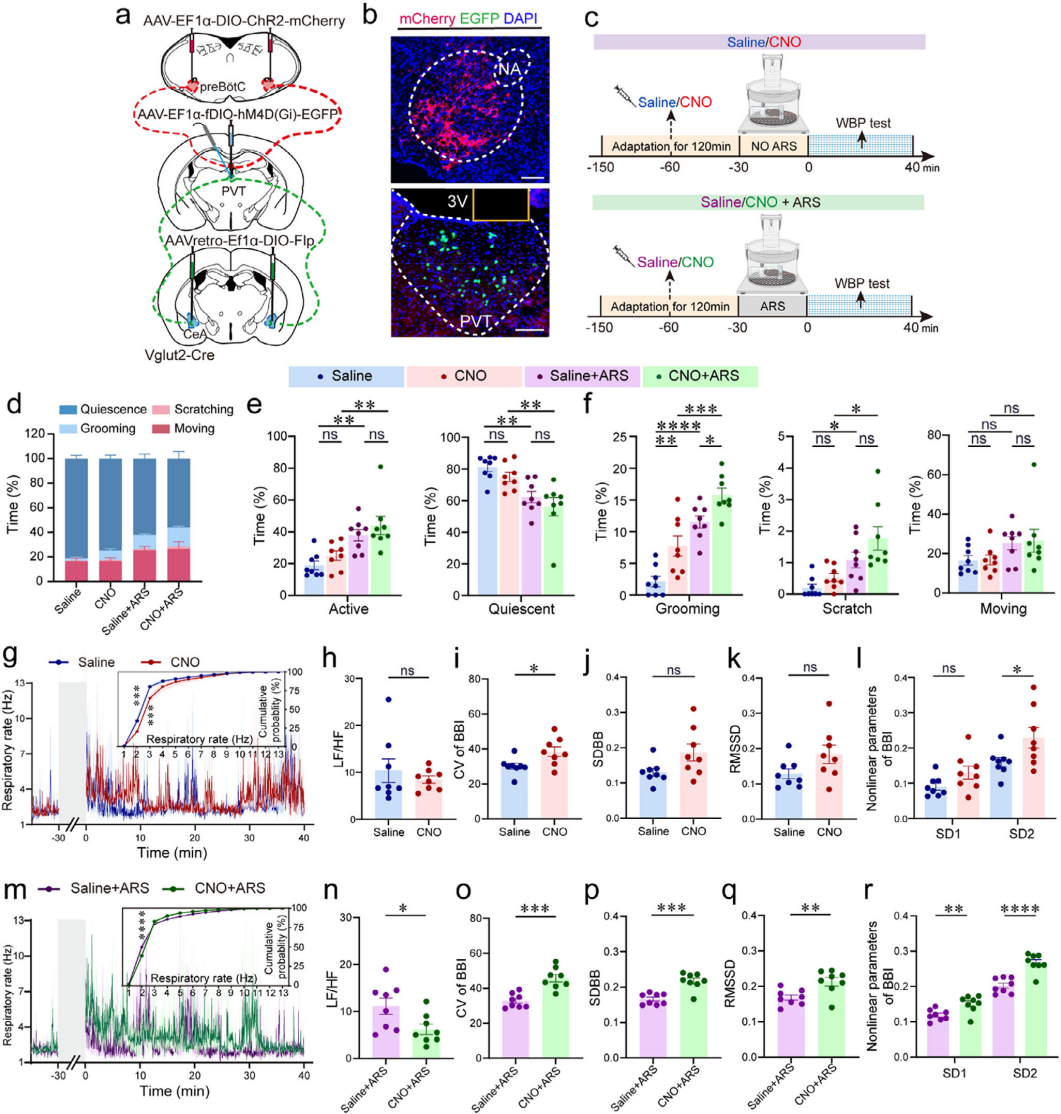
Inhibition of the PVT→CeA pathway abolishes the anxiolytic and respiratory benefits of preBötC^Glu^→PVT activation. (a) Viral strategy for concurrent optogenetic activation of the preBötC^Glu^→PVT pathway and chemogenetic inhibition of PVT→CeA projections. (b) Immunohistochemical validation of ChR2‑mCherry expression in the preBötC (top) and inhibitory construct in PVT neurons projecting to the CeA (bottom). Scale bars: 100 µm. (c) Experimental timeline for combined stimulation/inhibition under baseline (unrestrained) and ARS conditions. (d) Percentage of time spent in quiescent or active behaviors (grooming, scratching, moving) over a 40‑min recording. (e) Cumulative time in active versus quiescent states. (f) Proportion of total time devoted to each active behavior. (g) RF over time under baseline conditions. Inset: Cumulative RF distribution from mCherry‑control vs. ChR2‑mCherry‑expressing mice. (h) Ratio of LF to HF (LF/HF). (i–k) Quantitative analysis of CV (i), SDBB (j), and RMSSD (k) of BBI. (l) Nonlinear variability parameters (SD1, SD2) derived from Poincaré analysis. (m) RF over time following ARS. Inset: Cumulative RF distribution from ARS‑treated mCherry‑control vs. ChR2‑mCherry mice. (n) LF/HF ratio post‑ARS. (o–q) Quantitative analysis of CV (o), SDBB (p), and RMSSD (q) of BBI. (r) Nonlinear variability parameters (SD1, SD2) derived from Poincaré analysis post‐ARS. Sample sizes: *n* = 8 mice (d–f, h–l, n–r), = 7 (g, m) for each group. Statistical significance: All data are presented as the mean ± SEM. ^*^ *p* < 0.05, ^**^ *p* < 0.01, ^***^ *p* < 0.001, ^****^ *p* < 0.0001, as determined by two‐tailed unpaired *t*‐test (with Welch's correction where appropriate; h–l, n–r), one‐way ANOVA with Bonferroni's multiple comparisons tests (e,f) and two‐way ANOVA with Bonferroni's, Tamhane's T2 or Dunn's multiple comparisons tests (g,m).

To determine whether the PVT→CeA pathway serves as a critical downstream target of the anxiolytic effects elicited by preBötC^Glu^→PVT circuit activation, we performed loss‐of‐function assays via genetic strategies to selectively inhibit or ablate PVT neurons projecting to the CeA. First, we conducted chemogenetic inhibition experiments through delivering AAV‐EF1α‐fDIO‐hM4Di‐EGFP into the PVT, and injecting AAVretro‐EF1α‐DIO‐Flp into the CeA of the Vglut2‐Cre mice. Concurrently, AAV‐EF1α‐DIO‐ChR2‐mCherry was injected into the preBötC (Figure 5a), after which CNO (2 mg kg^−1^, i.p.) was administered to suppress hM4Di‐expressing neurons. Immunohistochemical analysis confirmed the expression of ChR2‐mCherry in the preBötC and hM4Di‐EYFP in the PVT (Figure 5b).

Behavioral experiments showed that, relative to the saline‐injected group, CNO administration significantly decreased both the number of entries into and time spent in the center field of the OFT (Figure S8a–c) as well as in the open arms of the EPM test in both ARS‐treated and unrestrained groups (Figure S8d,e). To further explore the impact of this inhibition on respiratory phenotypes, WBP recordings were carried out in both ARS‐treated and unrestrained mice (Figure 5c). Persistent photostimulation (473 nm, 10 mW, 20 ms pulse width, 10 Hz, 40 min) of the preBötC^Glu^→PVT circuit significantly increased grooming behavior in mice from the CNO group relative to the Saline group (Figure 5d–f), along with a marked elevation in RF and RF variability parameters (Figure 5g–r).

Next, we selectively ablated CeA‐projecting PVT neurons via targeted apoptotic ablation, as previously described [33]. This was achieved by delivering AAV‐EF1α‐fDIO‐taCasp3 or the control virus AAV‐EF1α‐fDIO into the PVT, and AAVretro‐flpo‐EGFP into the CeA of the Vglut2‐Cre mice. Concurrently, we introduced AAV‐EF1α‐DIO‐ChR2‐mCherry or the control virus AAV‐EF1α‐DIO‐mCherry into the preBötC (Figure S9a). Optical fibers were implanted into the PVT to enable optogenetic stimulation of the preBötC^Glu^→PVT circuit. Four weeks post‐injection, immunohistochemical analysis confirmed the ChR2‐mCherry expression in the preBötC and EGFP expression in the CeA and PVT (Figure S9b). We quantified EGFP‐expressing PVT neurons projecting to the CeA from both taCasp3‐injected mice and their counterparts. Quantitative analysis revealed a significant reduction in the number of EGFP‐expressing PVT neurons projecting to the CeA following ablation (Figure S9c). WBP (Figure S9d–u) and behavioral analyses (Figure S10a–e) demonstrated that, following genetic ablation, both the anxiolytic and respiratory phenotypes elicited by photostimulation of preBötC^Glu^ axon terminals within the PVT were markedly attenuated.

Collectively, chemogenetic inhibition or selective ablation of PVT neurons projecting to the CeA abolishes the anxiolytic effect of preBötC^Glu^→PVT circuit activation, suggesting that the PVT→CeA pathway is a critical downstream target of the preBötC^Glu^→PVT circuit in the regulation of anxiety‐like phenotypes.

### 2.6 PVT Neurons Projecting to the CeA Receive Monosynaptic Inputs From preBötC^Glu^ Neurons

**FIGURE 6:**
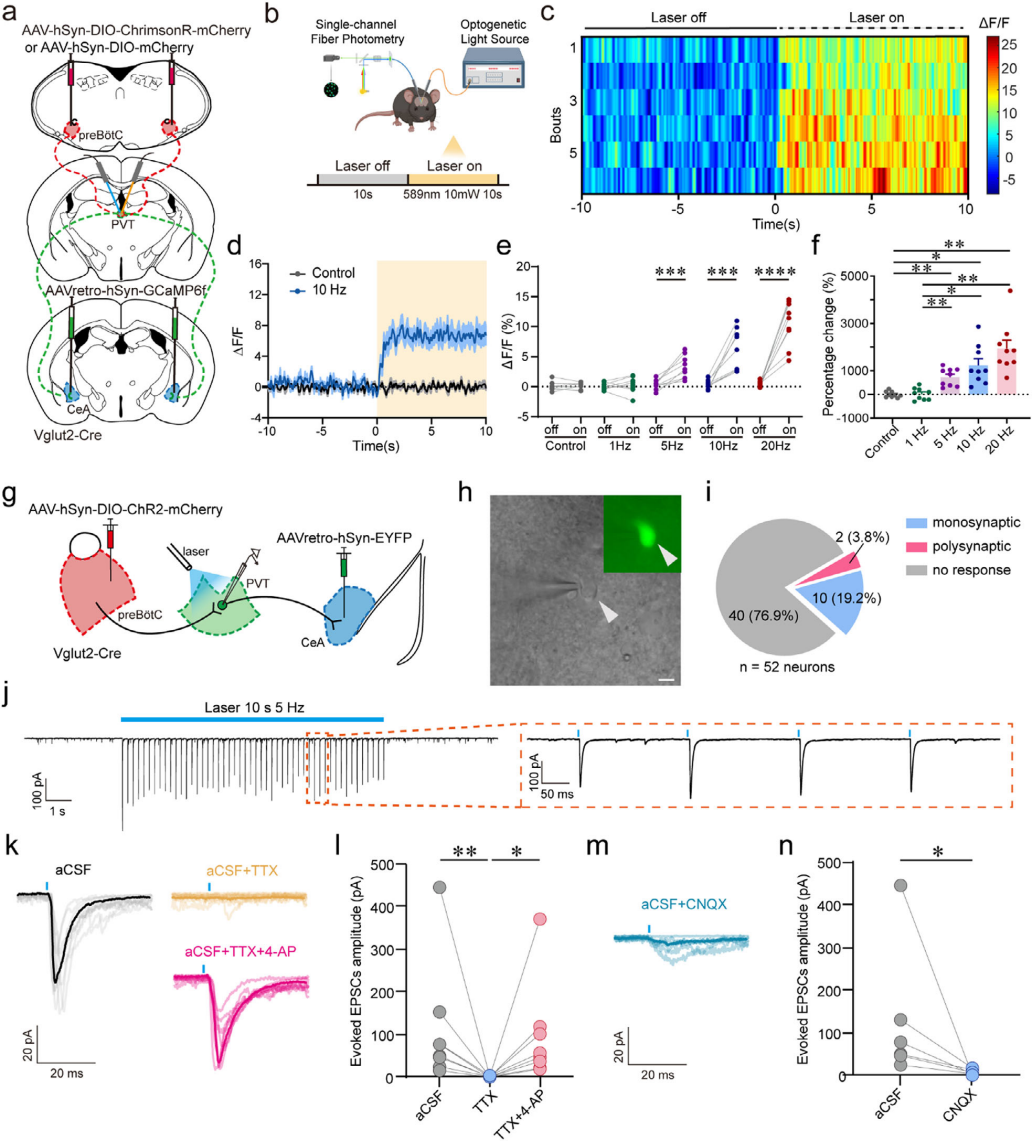
PVT neurons projecting to the CeA receive excitatory inputs from preBötC^Glu^ neurons. (a) Schematic of the viral injection strategy for photostimulation and Ca^2+^ signal imaging. (b) Schematic of the fiber photometry setup for recording GCaMP6f signals in PVT neurons. (c) Heatmap depicting Ca^2+^ activity in PVT neurons before and during photostimulation (10 Hz) of preBötC^Glu^ neuron axons within the PVT (*n* = 6 trials from 4 mice). (d) Example traces of photostimulation‐evoked Ca^2+^ signals in PVT neurons. (e,f) Quantitative analysis of calcium signal amplitude during photostimulation at different frequencies (*n* = 9 trials from 4 mice). (g) Experimental design for electrophysiological recording of excitatory postsynaptic currents (EPSCs) in retrogradely labeled PVT→CeA neurons during photostimulation of preBötC^Glu^ axons in the PVT. (h) Differential interference contrast (DIC) and fluorescence image of an EYFP‐labeled PVT neuron in a brain slice. Arrowheads indicate the recorded neuron. Scale bar, 10 µm. (i) Classification of synaptic connectivity from preBötC^Glu^ axons onto PVT→CeA neurons (*n*  =  52 neurons from 6 mice): monosynaptic (19.2%), polysynaptic (3.8%), or no response (76.9%). (j) Left: representative traces showing photostimulation (5 Hz)‐evoked EPSCs recorded in EYFP^+^ PVT neurons. Right: expanded view. (k–n) Pharmacology of evoked EPSCs: blockade by tetrodotoxin (TTX, 1 µM; *n*  =  8 neurons), rescue with 4‑aminopyridine (4‑AP, 100 µM; n  =  8 neurons), and complete inhibition by CNQX (10 µM; *n*  =  6 neurons). Statistical significance: All data are presented as the mean ± SEM. ^*^ *p* < 0.05, ^**^ *p* < 0.01, ^***^ *p* < 0.001, ^****^ *p* < 0.0001 by two‐tailed paired *t*‐test or Wilcoxon matched‐pairs signed rank test (e,n), Brown‐Forsythe and Welch ANOVA tests with Tamhane's T2 multiple comparisons tests (f), and Friedman test with Dunn's multiple comparisons test (l). Abbreviations: aCSF, artificial cerebrospinal fluid; TTX, tetrodotoxin; 4‐AP, 4‐aminopyridine; CNQX, 6‐cyano‐7‐nitroquinoxaline‐2,3‐dione.

To delineate functional connectivity between the preBötC^Glu^→PVT and PVT→CeA circuits, we integrated in vivo fiber photometry and in vitro electrophysiology. First, AAVretro‐hSyn‐GCaMP6f was delivered into the CeA to target PVT neurons projecting to the CeA, while a virus encoding the optogenetic actuator ChrimsonR (AAV‐hSyn‐DIO‐ChrimsonR‐mCherry) or a control virus (AAV‐hSyn‐DIO‐mCherry) was injected into the preBötC of Vglut2‐Cre mice (Figure 6a). Immunohistochemical validation confirmed GCaMP6f expression in the PVT and ChrimsonR‐mCherry expression in the preBötC (Figure S11a). We then monitored Ca^2+^ dynamics of PVT neurons projecting to the CeA in response to photostimulation (589 nm, 10 mW, 20 ms pulse width, 10 s) of preBötC^Glu^ neuron axon terminals within the PVT (Figure 6b). The heatmap and quantitative analysis reveal a sustained increase in Ca^2+^ signals during photostimulation (Figure 6c,d). Response amplitude increased in a frequency‐dependent manner (Figure 6e,f; Figure S11b–d). These findings demonstrate that stimulation of the preBötC^Glu^→PVT circuit enhances activation levels of the PVT→CeA circuit.

To further characterize the synaptic connections between the two circuits, we integrated photostimulation with whole‐cell patch clamp slice recordings. AAV‐EF1α‐DIO‐ChR2‐mCherry and AAVretro‐hSyn‐EYFP were correspondingly injected into the preBötC and CeA of Vglut2‐Cre mice (Figure 6g). Excitatory postsynaptic currents (EPSCs) were captured under a holding potential of –60 mV in EYFP‐expressing PVT neurons, while ChR2‐expressing axon terminals of preBötC^Glu^ neurons projecting to the PVT were illuminated (473 nm, 10 mW, 20 ms pulse width, 5 Hz, 10 s). A total of 52 neurons exhibited three response patterns: monosynaptic, polysynaptic and unresponsive (Figure 6h,i). In 10 of 52 (19.2%) neurons, EPSCs were effectively evoked by photostimulation at 5 Hz (Figure 6j), while they were blocked by bath application of the sodium channel blockers tetrodotoxin (TTX) but restored by administration of the potassium channel blocker 4‐aminopyridine (4‐AP) (Figure 6k,l), confirming monosynaptic connectivity. In 2 of 52 (3.8%) neurons, TTX abolished photostimulation‐evoked EPSCs without reversal by 4‐AP, suggesting polysynaptic connectivity. The remaining 40 (76.9%) neurons were unresponsive. Consistent with previous findings [34] and the glutamatergic nature of most PVT neurons, photostimulation‐evoked EPSCs were blocked by the glutamate receptor antagonist 6‐cyano‐7‐nitroquinoxaline‐2,3‐dione (CNQX) (Figure 6m,n). In summary, we demonstrate that preBötC^Glu^→PVT axons form monosynaptic excitatory connections with PVT→CeA‐projecting neurons, providing a physiological basis for the functional integration of these circuits.

### 2.7 Divergent PVT Projections to CeA Subnuclei Mediate Bidirectional Control of Anxiety via a preBötC‐Initiated Circuit

**FIGURE 7:**
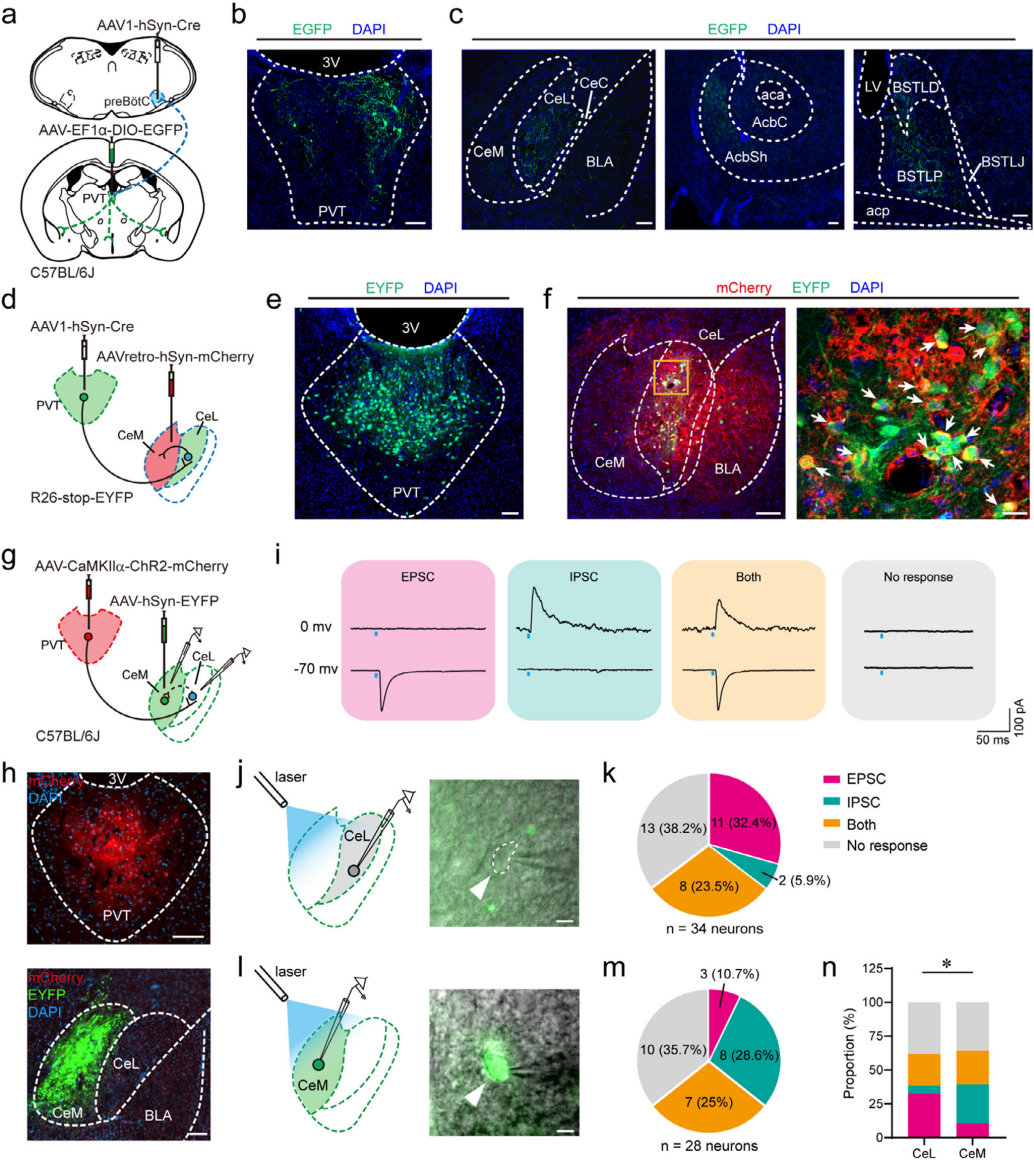
Divergent modulation of CeL and CeM neurons by PVT inputs. (a) Strategy for anterograde transsynaptic tracing from the preBötC to postsynaptic targets using AAV1‑hSyn‑Cre. (b) Immunostaining showing EGFP^+^ PVT neurons innervated by preBötC inputs. Scale bar, 100 µm. (c) Distribution of axon terminals from the labeled PVT neurons, showing dense innervation of the CeA, NAc and BNST. Scale bars, 100 µm. (d) Intersectional viral strategy in R26‑stop‑EYFP mice to simultaneously label PVT‑innervated CeL neurons and CeM‑projecting neurons. (e) Representative image of EYFP^+^ PVT neurons. Scale bar, 100 µm. (f) Low‑ (left) and high‑magnification (right; boxed region) views showing EYFP^+^ (PVT‑innervated) neurons, mCherry^+^ (CeM‑projecting) neurons, and double‑labeled EYFP^+^mCherry^+^ neurons in the CeL. Scale bars: 100 µm (left), 20 µm (right). (g) Schematic of the combined viral and electrophysiological approach to assess synaptic connectivity between PVT axons and CeA subnuclei. (h) Top: ChR2‑mCherry expression in the PVT. Bottom: EYFP^+^ CeM neurons and ChR2‑mCherry^+^ PVT axon terminals in the CeL. Scale bars, 100 µm. (i) Raw traces showing photostimulation (473 nm, 10 mW, 10 ms pulse width) of axonal terminals in the CeA evoked EPSCs and IPSCs recorded in CeL neurons. Each colored region showing evoked EPSCs, IPSCs or both in the same neuron under different holding potentials. (j) Left: Schematic of recording configuration in the CeL. Right: Differential interference contrast (DIC) image of a patched CeL neuron. Scale bars, 10 µm. (k) Proportion of CeL neurons (*n*  =  34 from 8 mice) exhibiting excitatory, inhibitory, mixed, or no responses to PVT axon stimulation. (l) Left: Schematic of recording configuration in the CeM. Right: DIC image of a patched, fluorescently labeled CeM neuron. Scale bars, 10 µm. (m) Proportion of CeM neurons (*n*  =  28 from 8 mice) exhibiting different response types to PVT stimulation. (n) Response profile proportions of CeL versus CeM neurons to PVT stimulation. Statistical significance: ^*^p < 0.05 by Fisher's exact test (n). Abbreviations: aca, anterior commissure, anterior part; AcbC, nucleus accumbens, core region; AcbSh, nucleus accumbens shell; acp, anterior commissure, posterior limb; BSTLD, bed nucleus of the stria terminalis, lateral division, dorsal part; BSTLP, bed nucleus of the stria terminalis, lateral division, posterior part; BSTLJ, bed nucleus of the stria terminalis, lateral division, juxtacapsular part; CeL, central amygdaloid nucleus, lateral part; CeM, central amygdaloid nucleus, medial part; CeC, central amygdaloid nucleus, capsular part; LV, lateral ventricle.

Emerging evidence indicates that activation of GABAergic neurons in the CeA drives anxiety‐like phenotypes through the recruitment of the CeA‐PVT circuit [21]. Here, we demonstrate that activation of the preBötC‐PVT‐CeA circuit alleviates anxiety‐like phenotypes via a feedback mechanism involving CeA neurons. However, the precise mechanisms by which the PVT regulates local circuits within the CeA remain a critical challenge. To address this, we employed an anterograde transsynaptic tracing approach by injecting AAV1‐hSyn‐Cre into the preBötC and AAV‐EF1α‐DIO‐EGFP into the PVT of C57BL/6J mice (Figure 7a). Four weeks post‐injection, immunohistochemical staining identified EGFP‐expressing PVT neurons as postsynaptic targets of preBötC neurons (Figure 7b). Moreover, axons of these EGFP^+^ PVT neurons were detected in several brain regions, including the CeA, nucleus accumbens (NAc) and the bed nucleus of the stria terminalis (BNST) (Figure 7c). Notably, these axons were predominantly distributed within the centrolateral amygdala (CeL), with minimal presence in the centromedial amygdala (CeM).

Previous studies have reported that the CeL provides inhibitory input to the CeM [35] and that activation of CeM neurons induces anxiety‐like phenotypes [36]. Given these findings, we hypothesized that stimulation of PVT neurons innervated by the preBötC attenuates anxiety‐like phenotypes by acting on CeL neurons, thereby inhibiting CeM neurons. To test this hypothesis, AAV1‐hSyn‐Cre was delivered into the PVT of R26‐stop‐EYFP mice, while the retrograde tracing virus AAVretro‐hSyn‐mCherry was injected into the unilateral CeM (Figure 7d). Immunohistochemical analysis demonstrates numerous EYFP^+^ neurons in the PVT (Figure 7e), with some identified in the CeL and minimal presence in the CeM (Figure 7f), suggesting that PVT neurons primarily target CeL rather than CeM neurons. Meanwhile, a number of mCherry^+^ neurons targeting the CeM was detected in the CeL. Then, the EYFP^+^mCherry^+^ CeL neurons likely serve as relay interneurons between the PVT and CeM. Immunohistochemical analysis revealed that EYFP^+^mCherry^+^ neurons were predominantly located within the CeL (Figure 7f). Collectively, these findings demonstrate that the PVT establishes synaptic connection with the CeM via CeL interneurons.

To delineate the functional synaptic connectivity among the PVT, CeL and CeM, we conducted whole‐cell patch clamp recordings in brain slices. C57BL/6J mice received injections of AAV‐CaMKIIα‐ChR2‐mCherry into the PVT and AAV‐hSyn‐EYFP into the CeM (Figure 7g). Immunohistochemical validation confirmed the presence of mCherry^+^ neurons in the PVT (predominantly glutamatergic), mCherry^+^ axon terminals in the CeL and EYFP^+^ neurons in the CeM (Figure 7h). We examined whether EPSCs and IPSCs recorded in CeL neurons could be evoked by photostimulation (473 nm, 10 mW, 10 ms pulse width) of PVT neuron axon terminals within the CeA. Of 34 CeL neurons recorded, 32.4% (*n* = 11) exhibited evoked EPSCs only; 5.9% (*n* = 2) showed IPSCs only; 23.5% (*n* = 8) displayed both EPSCs and IPSCs; 38.2% (*n* = 13) were unresponsive (Figure 7i–k). These findings indicate that stimulation of PVT neurons preferentially provides excitatory drive to CeL neurons (P_excitation_ = 58.8% vs. P_inhibition_ = 35.3%), irrespective of mono‐ or polysynaptic connections. A similar experimental approach was applied to CeM neurons. In EYFP‐expressing CeM neurons tested (*n* = 28), 10.7% (*n* = 3) exhibited photostimulation‐evoked EPSCs only, with IPSCs only in 28.6% (*n* = 8), both EPSCs and IPSCs in 25% (*n* = 7), and no response in 35.7% (*n* = 10) (Figure 7l,m). The bias toward inhibition (P_inhibition_ = 57.1% vs. P_excitation_ = 35.7%) implies CeL‐mediated suppression of CeM activity following PVT stimulation. Statistical analysis further confirmed a significant difference in the response profiles of CeL vs. CeM neurons to PVT stimulation, with excitation predominating in the CeL and inhibition predominating in the CeM (Figure 7n). Collectively, these results suggest that activation of PVT neurons preferentially gates CeM activity likely via acting on CeL^GABA^ neurons, providing mechanistic insights into the divergent modulation of CeA subdivisions by the PVT.

**FIGURE 8:**
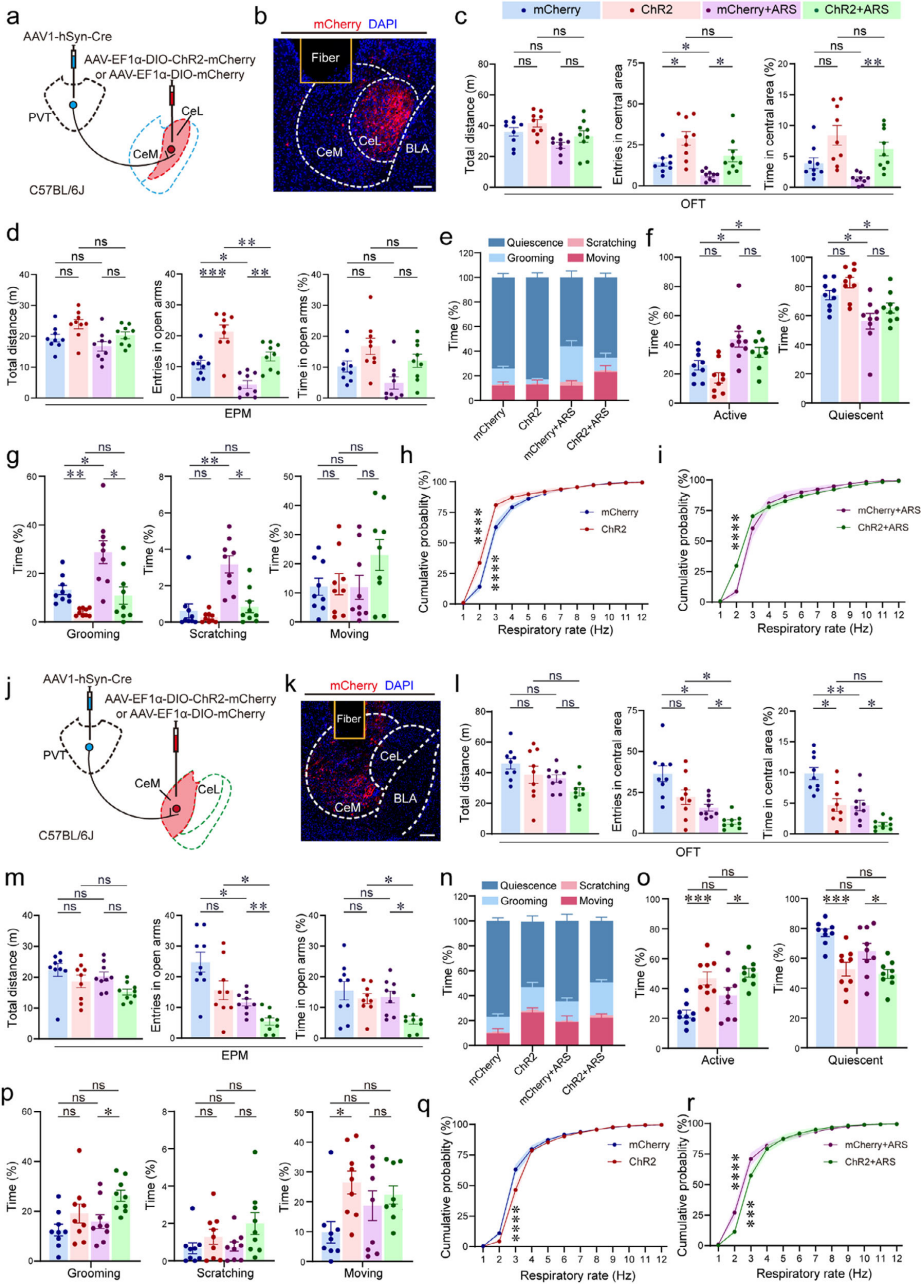
PVT→CeL and PVT→CeM pathways bidirectionally modulate anxiety‐like phenotypes and respiratory dynamics. (a) Optogenetic viral strategy to target PVT axon terminals within the CeL. (b) Representative immunohistochemistry showing mCherry^+^ CeL neurons receiving PVT projections. Scale bar, 100 µm. (c,d) Anxiolytic effects of PVT→CeL photostimulation, determined by quantitative analysis parameters of OFT and EPM tests. (e) Distribution of time spent in specific states (quiescence, grooming, scratching, moving) during a 40‐min recording. (f) Accumulative time spent in an active vs. quiescent state. (g) Proportion of total time allocated to each active behavior. (h,i) Cumulative distribution of RF under baseline (h) and post‐ ARS (i) conditions in CeL‐targeted mice. (j) Viral strategy for targeting PVT axon terminals in the CeM. (k) Confirmation of mCherry^+^ CeM neurons receiving PVT inputs. Scale bar, 100 µm. (l,m) Anxiogenic effects of PVT→CeM photostimulation, demonstrated by quantitative analysis of parameters of OFT and EPM tests. (n) Distribution of time spent in quiescence, grooming, scratching, and moving during photostimulation over a 40‐min recording. (o) Cumulative time in active vs. quiescent states during CeM stimulation. (p) Percentage of time spent in each active behavior. (q,r) Cumulative RF distribution under baseline (q) and post‐ARS (r) conditions in CeM‐targeted mice. Sample sizes: *n* = 9 mice per group (c–g, l–p), = 6 (h, i, q, r). Statistical significance: All data are presented as the mean ± SEM. ^*^ *p* < 0.05, ^**^ *p* < 0.01, ^***^ *p* < 0.001, ^****^ *p* < 0.0001, as determined by one‐way ANOVA with Bonferroni's multiple comparisons tests (c, d, f, g, l, m, o, p) and two‐way ANOVA with Bonferroni's, Tamhane's T2 or Dunn's multiple comparisons tests (h, i, q, r).

To delineate the behavioral functions of PVT projections to distinct CeA subnuclei, we selectively manipulated the PVT→CeL and PVT→CeM circuits using optogenetics combined with behavioral analysis and WBP. To achieve these conditions, C57BL/6J mice received injections of AAV1‐hSyn‐Cre into the PVT, followed by injection of either AAV‐EF1α‐DIO‐ChR2‐mCherry or the control virus AAV‐EF1α‐DIO‐mCherry into the CeL/CeM, respectively (Figure 8a,j).  Histological analysis confirmed restricted opsin expression within the targeted CeA subdivisions (Figure 8b,k).

Photostimulation (473 nm, 10 mW, 20 ms pulse width, 10 Hz, 40 min) of the PVT→CeL circuit produced a pronounced anxiolytic effect. ChR2‐expressing mice exhibited a significant increase in both entries into and time spent in the center of the open field (Figure 8c) and the open arms of the EPM (Figure 8d), compared to mCherry controls. Furthermore, these mice showed reduced durations of self‐directed grooming and scratching behaviors, alongside a decrease in RF, under both ARS and baseline conditions (Figure 8e–i). Conversely, photostimulation of the PVT→CeM pathway evoked robust anxiogenic responses. ChR2‐expressing mice spent significantly less time in, and made fewer entries into, the anxiogenic zones of the OFT and EPM (Figures 8l,m). This was accompanied by increased active time, decreased quiescent time, and an accelerated RF in both ARS and unrestrained settings (Figure 8n–r).

Collectively, these results demonstrate that the PVT exerts bidirectional control over anxiety‐like states via topographically segregated projections to the CeA: the PVT→CeL circuit suppresses, while the PVT→CeM circuit promotes, anxiety‐like phenotypes and physiological responses.

### 2.8 Slow Breathing Attenuates Anxiety by Modulating Amygdala Oscillatory Activity in Human

**FIGURE 9:**
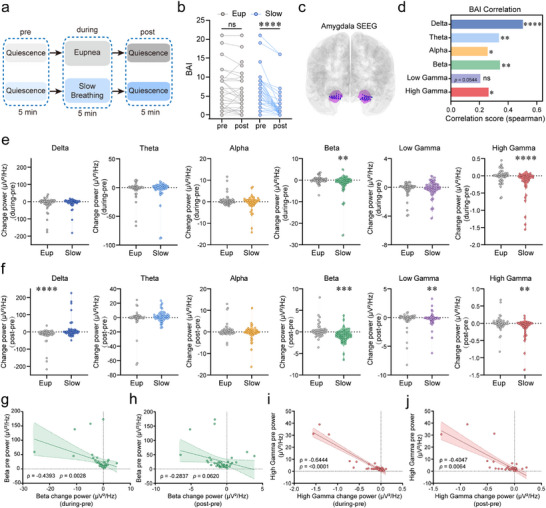
Volitional slow breathing reduces subjective anxiety and suppresses pathological amygdala oscillations in humans. (a) Experimental design: healthy volunteers (*n* = 78) were assigned to either volitional slow breathing (4–7 breaths min^−1^) or eupneic breathing (12–20 breaths/min) for 5 min. (b) Slow breathing significantly reduced Beck Anxiety Inventory (BAI) scores relative to eupneic breathing (*n* = 39 per group). (c) Schematic illustrating the analysis of 42 bipolar channel signals derived from 54 intracranial contacts within the amygdala of epilepsy patients (*n* = 12). (d) Correlations between pre‐intervention BAI scores and absPSD across delta, theta, alpha, beta, and high‐gamma frequency bands during eupneic breathing. Positive correlations were observed across all frequency bands. (e) Slow breathing significantly suppressed the power of beta and high‐gamma oscillations, measured as the difference between the during‐intervention and pre‐intervention baseline. (f) Slow breathing significantly suppressed the power of beta, low‐gamma, and high‐gamma oscillations, measured as the difference between post‐intervention and pre‐intervention baseline. (g–j) Relationship between baseline (pre‐intervention) power levels of beta and high‐gamma oscillations and their changes during and post‐intervention. Baseline beta power was negatively correlated with the power changes during the intervention (during/pre) (g), but not with the change post‐intervention (post/pre) (h). In contrast, baseline high‐gamma power showed a negative correlation with both during‐intervention (during/pre) (i) and post‐intervention changes (post/pre) (j). Statistical significance: ^*^ *p* < 0.05, ^**^ *p* < 0.01, ^***^ *p* < 0.001, ^****^ *p* < 0.0001, as determined by two‐tailed paired *t* test (e,f) and Spearman test (d, g–j). All data are presented as the mean ± SEM. Abbreviations: absPSD, absolute power spectral density; BAI, Beck Anxiety Inventory. Eup, eupnea.

Substantial evidence indicates that slow, rhythmic breathing mitigates anxiety, yet the underlying neural mechanisms remain undefined. Building on our demonstration that the preBötC→PVT→CeA circuit activation alleviates ARS‐induced anxiety‐like phenotypes and respiratory dysregulation in mice, we investigated slow breathing's effects in humans. Healthy volunteers (*n* = 78) were divided into matched cohorts: one undergoing volitional slow breathing (4–7 breaths/min), while the other maintained eupneic breathing (12–20 breaths/min) for 5 min (*n* = 39 per group; Figure 9a). Beck Anxiety Inventory (BAI) assessment revealed a significant reduction in anxiety levels following slow breathing compared to eupneic controls (Figure 9b).

To explore the neural correlates of this phenomenon, we analyzed intracranial recordings from amygdala‐targeting electrodes in epilepsy patients (*n* = 12; 54 contacts, 42 bipolar channels; Figure 9c). According to the BAI scores and the absolute power spectral analysis (absPSD), baseline anxiety levels (pre‐intervention BAI scores) exhibited positive correlations with absolute oscillatory power across delta, theta, alpha, beta, and high‐gamma frequency bands (Figure 9d), suggesting that amygdala activity spanning a broad frequency range is associated with anxiety. Importantly, volitional slow breathing selectively suppressed beta and high‐gamma oscillations, measured as the difference between during‐intervention and pre‐intervention baseline (Figure 9e). Furthermore, we demonstrated a marked suppression of changes in the power of beta, low‐gamma, and high‐gamma oscillatory activity between pre‐ and post‐intervention phases (Figure 9f), indicating both immediate and prolonged effects of slow breathing on amygdala activity. We also examined the relationship between baseline oscillatory power and intervention‐induced changes. Baseline beta power levels negatively correlated with power changes during the intervention (during/pre) but not post‐intervention (post/pre) (Figure 9g,h). In contract, baseline high‐gamma power levels negatively correlated with both during‐intervention changes (during/pre) and post‐intervention changes (post/pre) (Figure 9i,j).

These findings demonstrate that slow breathing specifically modulates beta and high‐gamma oscillatory activity in the amygdala, with baseline activity levels influencing the extent of these changes. Together, these results provide mechanistic insights into how slow breathing attenuates anxiety, highlighting the amygdala as a key neural substrate mediating these effects.

## 3 Discussion

In this study, we define the preBötC^Glu^→PVT→CeA circuit as a conserved mediator of breathing‐emotion integration. In male mice, activation of the preBötC^Glu^→PVT circuit alleviates ARS‐induced anxiety‐like phenotypes and normalizes RF and RF variability. Conversely, inhibiting this circuit exacerbates anxiety‐like phenotypes and respiratory dysfunction. The anxiolytic and respiratory effects of pathway activation are contingent upon PVT→CeA projections, as their inhibition or ablation significantly attenuates these phenotypes. We further demonstrate that this modulation is executed via divergent PVT outputs: whereas PVT→CeL projections mediate anxiolysis, PVT→CeM projections promote anxiogenic responses. Translating these preclinical insights, we show in humans that volitional slow breathing reduces subjective anxiety in healthy individuals and suppresses anxiety‐related beta/high‐gamma oscillatory activity within the amygdala of epilepsy patients. Collectively, these cross‐species findings delineate a conserved thalamo‐amygdalar circuit that integrates respiratory control with affective state modulation.

### 3.1 Specific Respiratory Phenotypes as Potential Biomarkers of Anxiety

Anxiety disorders encompass core psychobehavioral symptoms—excessive worry, psychomotor agitation, and sleep disturbances—frequently accompanied by autonomic dysregulation. This dysregulation manifests as cardiovascular instability and pronounced respiratory abnormalities, including pathologic sighing and panic‐associated hyperventilation [7, 37]. While heart rate variability is an established biomarker for anxiety [38], respiratory phenotypes remain underexplored for the clinical stratification of anxiety states.

Growing evidence indicates that emotional states are encoded by distinct respiratory signatures across both animal models and humans [26, 27, 39, 40]. Leveraging established rodent anxiety assessments, such as the OFT and EPM, we integrated WBP with videography to simultaneously capture behavioral states and respiratory dynamics. This multimodal approach revealed that anxiety‐like phenotypes correlate with active behaviors (e.g., grooming, scratching, moving) characterized by high‐frequency breathing bouts, in contrast to low‐variability eupnea of quiescent states [12, 21, 29, 41]. Extending beyond heart rate variability, we identify RF variability—quantified via time‐domain and nonlinear Poincaré analysis of BBI—as a potential objective biomarker for anxiety.

Here, we employed the well‐established ARS paradigm to induce anxiety‐like phenotypes in mice [19, 42]. Mechanistically, our ARS model links stress‐induced anxiety to respiratory dysregulation. Initially, OFT and EPM test analyses confirmed the induction of anxiety‐like phenotypes. Subsequent quantitative assessment of WBP and videography demonstrated behavioral dysregulation in ARS‐treated mice characterized by prolonged active states and reduced quiescence compared to unrestrained controls. These active states were associated with high‐frequency breathing bouts and decreased LF/HF ratio. Meanwhile, we introduced RF variability analysis to evaluate anxiety‐like phenotypes. Crucially, RF variability analysis demonstrated respiratory dysrhythmia in ARS‐treated mice, marked by increased high‐frequency breathing bouts, elevated CV of BBI, and amplified short‐term respiratory instability (increased Poincaré SD1 with unchanged SD2). Together, these convergent behavioral and respiratory signatures establish a robust quantitative framework for modeling anxiety‐like phenotypes in rodents.

Although time‐domain, frequency‐domain, and nonlinear analyses have been applied to assess respiratory variability in mechanically ventilated patients [43, 44], respiratory biomarkers remain largely unexplored in psychiatric disorders. Our findings establish RF variability as an objective, mechanistically grounded indicator of anxiety states in animal models. This discovery holds significant translational potential, warranting clinical validation in human populations to advance the development of respiratory‐based biomarkers for anxiety disorders.

### 3.2 Thalamic Integration of Respiratory‐Limbic Signals

Volitional breathing practices, rooted in ancient spiritual traditions such as yoga and Tai Chi, have long been employed to rebalance emotions and mitigate stress [7]. However, the neural mechanisms underlying these effects remains poor defined. Recent studies have identified a preBötC→LC circuit that mediates the balance between calm and arousal behaviors [12]. Subsequent investigation has demonstrated that a dorsal anterior cingulate cortex→pontine reticular nucleus→ventrolateral medulla circuit exerts a top‐down control of breathing, thereby influencing emotional states [11]. These findings underscore the profound capacity of respiratory rhythm to influence affective processing.

The preBötC, a key medullary site for respiratory rhythmogenesis [10], integrates monosynaptic inputs from diverse brain regions, including the brainstem, thalamus, hypothalamus, and cortex [45]. This broad connectivity positions it to modulate breathing patterns in response to multisystemic signals [21]. In turn, preBötC neurons, including somatostatin (SST)‐expressing and glycinergic populations, project reciprocally to the brainstem, hypothalamus, thalamus, and limbic targets [46], forming bidirectional loops for physiological regulation. We have previously demonstrated that preBötC^SST^ neuron activation drives a slow, deep breathing pattern via dual innervation of Phox2b^+^ and GABAergic neurons in the NTS [47]. Concurrently, the amygdala has been identified as a crucial hub for processing emotions, as reported by both clinical [14, 15] and animal studies [16]. In particular, the CeA serves as the primary output region, playing an essential role in modulating anxiety‐like phenotypes and the development of anxiety disorders [17, 18]. Synthesizing these observations, we hypothesized the existence of a dedicated circuit integrating respiratory rhythm generation with anxiety states.

First, we anatomically defined a tripartite circuit comprising preBötC^Glu^→PVT^Glu^→CeA. The PVT's role as an anxiety‐processing hub was recognized, but its respiratory integration remained unexplored. The PVT was selected as a critical relay node due to the high proportion of its neurons that are both activated by preBötC inputs and project to the CeA, outperforming other candidate nuclei (e.g., NTS, LC, PBN). Additionally, the PVT is a recognized integrative hub within anxiety networks [48], with major outputs to the amygdala, BNST and NAc. Previous studies have indicated that PVT modulates the learning of fear memories, particularly through interactions with the CeL [49]. Furthermore, the PVT–CeA circuit has been implicated in regulating hyperarousal in response to acute stress [42]. Collectively, in combination with prior studies, we demonstrate that preBötC^Glu^ inputs preferentially excite PVT^Glu^ neurons, thereby providing a direct anatomical link between brainstem respiratory centers and limbic emotional processors.

Second, we established the functional necessity of this circuit in modulating anxiety‐like phenotypes. Respiratory rhythm is highly coupled with brain activity, providing a substrate for influencing behaviors and emotions. Slow breathing, specific breathing patterns (e.g., prolonged exhalation or box breathing), and mindfulness practices have been shown to effectively manage emotions [50, 51, 52]. Our findings reveal that inhibition of the preBötC^Glu^→PVT circuit exacerbated ARS‐induced anxiety‐like phenotypes and respiratory dysfunction, whereas circuit activation produced robust anxiolytic and respiratory‐stabilizing effects. These benefits were abolished by inhibiting or ablating PVT^Glu^ neurons projecting to the CeA, confirming pathway specificity. Mechanistically, circuit activation likely normalizes RF and RF variability by rebalancing behavioral states, suppressing high‐frequency breathing (4‒7 Hz) linked to active behaviors (e.g., grooming, scratching) and promoting the low‐variability eupnea of quiescence (2‒4 Hz).

Third, we resolved a key paradox: how can PVT activation be anxiolytic when CeA^GABA^→PVT circuit activation is anxiogenic? [21] The CeA features specialized microcircuits for negative valence processing [53]. For example, PVT neurons project extensively to the CeL [54], where CeL^GABA^ neurons inhibit CeM^GABA^ neurons [55], potentially promoting a disinhibitory effect on downstream targets. In the present study, we provide anatomical, electrophysiological, and functional evidence that PVT inputs preferentially target GABAergic neurons in the CeL, which subsequently inhibit GABAergic outputs from the CeM. This CeL→CeM disinhibitory motif likely suppresses downstream anxiogenic pathways, explaining the anxiolytic outcome of PVT stimulation. This model aligns with observations that BLA inputs to the CeL also reduce anxiety [56].

While we establish the PVT→CeA pathway as a dominant mediator, parallel preBötC→PVT projections to the BNST and NAc—both implicated in anxiety [27, 57, 58] —may also contribute to the affective benefits of controlled breathing. Future work should delineate the role of these parallel pathways and examine CeA subnuclei dynamics during respiratory‐entrained neural oscillations.

### 3.3 Slow Breathing Attenuates Anxiety via Respiratory‐Limbic Circuit Integration

Accumulating evidence positions respiration as a potent modulator of brain‐wide signaling [59, 60], with breathing‐locked neural oscillations increasingly implicated in motor [61], cognitive [62], and perceptual processes [63]. This respiratory modulation represents an evolutionarily conserved anxiolytic strategy, though its underlying neural circuitry remains incompletely defined. Clinically, controlled breathing techniques that modify rate and depth have proven effective in mitigating negative emotional states such as anxiety and depression [64]. In this study, we demonstrate that voluntary slow breathing reduces anxiety in healthy individuals, effectively phenocopying the anxiolytic effects of chemogenetic circuit activation in mice. Moreover, in epilepsy patients, slow breathing selectively suppresses anxiety‐related beta/high‐gamma oscillatory activity in the amygdala, a frequency‐dependent modulation that parallels neuronal firing changes observed in the rodent CeA. This cross‐species convergence points to a conserved glutamatergic axis that tunes emotional states through respiratory feedback.

Our previous work identified CeA→PVT and CeA→LPBN→preBötC as distinct mediators of anxiety‐like phenotypes and high‐frequency breathing bouts, respectively, suggesting a top‐down control of respiration [21]. The present study shows that activation of the preBötC→PVT→CeA circuit significantly attenuates ARS‐induced anxiety levels, while reducing RF and RF variability. The observed decrease in RF is likely attributable to a shift from active to quiescent behavioral states, though alternative mechanisms may coexist. For example, preBötC^Glu^ neurons may influence breathing via collateral projections to local circuits and distal targets like the PVT. Additionally, activation of glutamatergic preBötC^SST^ neurons projecting to the NTS induces deep and slow breathing [47], suggesting that a subset of preBötC^Glu^ neurons could exert a feedback control of breathing through projecting to distal targets, including the PVT and LC. Although activation of the preBötC→PVT→CeA circuit reduces RF and RF variability involuntarily, it establishes a concrete mechanistic link between respiratory rhythmogenesis and emotional processing.

This study was conducted in male mice. Although our previous findings show that ARS elicits similar anxiety‐like phenotypes in males and females [21], extending this circuit analysis to females is a necessary future direction to comprehensively define the role of respiratory‐limbic pathways in anxiety, particularly given the known sexual dimorphism in its clinical presentation.

### 3.4 Summary

In summary, the anatomical and functional elucidation of the preBötC→PVT→CeA circuit re‐conceptualizes ancient breathing practices from empirical behavioral traditions into a mechanism‐based, circuit‐level intervention (Figure S12). This work establishes respiratory rhythm as a physiologically tunable entry point for modulating anxiety states, providing a mechanistic roadmap for developing targeted bioelectronic strategies. Therapeutically, our findings validate slow breathing not as a mere placebo but as a form of endogenous, circuit‐specific neuromodulation. Looking forward, biofeedback technologies could utilize real‐time respiratory frequency variability as a quantifiable biomarker to personalize and guide interventions for anxiety and related neuropsychiatric conditions.

## 4 Experimental Section

### 4.1 Animals

C57BL/6J (Beijing Vital River Laboratory Animal Technology, Beijing, China), R26‐stop‐EYFP (Jackson Laboratory stock: JAX006148), Vglut2‐Cre (Jackson Laboratory stock: JAX016963), and Vgat‐Cre (Jackson Laboratory stock: JAX016962) mice were used in this experiment. The experiments were conducted in male mice (8–12 weeks). The mice were housed under program‐controlled temperature (24 ± 1°C) and humidity (50 ± 10%) with a fixed 12 h: 12 h light: dark cycle (beginning at 7:00 a.m.) with ad libitum access to food and water. Age‐balanced littermate mice were randomly assigned to the experimental groups. All behavioral tests were conducted during the light phase. In the behavioral tests, the mice were allowed to adapt to laboratory conditions for approximately 1 week and habituate to the testing situation for at least 2 h before the experiments. Biohazard waste was managed in accordance with the Medical Waste Management Regulations of China. All experiments were performed in accordance with the Guide for the Care and Use of Laboratory Animals and approved by the Animal Care and Ethical Committee of Hebei Medical University.

### 4.2 Human Participants

Both health volunteers and epilepsy patients were recruited for this study. Health volunteers aged between 18 and 40 years of both sexes were recruited. The exclusion criteria were presented as follows: (1) under 18 years of age; (2) without education; (3) with a history of psychiatric illness; (4) using psychotropic drugs (including SSRIs). Finally, a total of 78 health volunteers (39 males, 39 females; mean 27.54 ± 5.99 years old) were recruited. A total of 27 epilepsy patients with normal intelligence (MoCA > 20) and undergoing presurgical stereo‐electroencephalogram (SEEG) were recruited, but 15 of the epilepsy patients were excluded due to no electrodes or excessive inter‐epileptic discharges in the amygdala. The final recruited 12 epilepsy patients were 7 male and 5 female with an average age of 30.7 ± 7.8 years old. They were implanted with multiple intracranial depth electrodes (Huake Hengsheng, Beijing, China, or Alcis, Besancon, France) to monitor brain activity, in order to determine the exact location of the epileptic foci. Intracranial electrode placements were entirely determined by clinical considerations. All procedures were conducted in accordance with the ethical standards of the Institutional Research Committee and the Declaration of Helsinki and its subsequent amendments (1964) or comparable ethical standards. Approval for all procedures was obtained from the Ethics Committee of Ruijin Hospital, School of Medicine, Shanghai Jiao Tong University (approval number: 2024–276) and the Ethics Committee of Zhongshan Hospital, School of Medicine, Shanghai Jiao Tong University (Approval Number: B2025‐249), and registered at chictr.org.cn website (ChiCTR2500098543). In accordance with the Declaration of Helsinki, informed consent was obtained from all participants through their written endorsement.

### 4.3 Acute Restraint Stress Model

The protocol for ARS treatment has been detailed previously [21]. In brief, mice were restrained using a small transparent tube (3 cm diameter, 10 cm length) with a hole in the top of the tube. For behavioral tests, the ARS‐treated mice were subjected to 30 min of complete physical confinement within restraint tubes, while the unrestrained mice were exposed to their home cages containing a restraint tube for the same duration, with no confinement. After restraint or unrestrained treatment, all mice remained in their home cage for 0, 30, or 60 min and were then subjected to behavioral tests. Similarly, for breathing tests, ARS‐treated mice were gently immobilized in the restraint tube and kept in the test chamber for 30 min. Unrestrained mice were exposed to a restraint tube within the same test chambers for 30 min, but were allowed free movement, as they were not immobilized. Throughout these procedures, mice were continuously monitored to ensure proper ventilation and well‐being.

### 4.4 Breathing Measurements

All protocols were performed as previously described [21]. The ventilatory response was measured using whole‐body plethysmography (EMKA Technologies; DSI/Buxco). The mass flow regulator provided a quiet, constant, and smooth air mixture through the animal chamber (0.5 L min^−1^) maintained at room temperature. The chamber temperature and humidity were continuously monitored and used to correct the tidal volume on a breath‐by‐breath basis. To measure the response of post‐stress breathing patterns and ventilatory parameters in mice, the animals were allowed to acclimate in the chamber individually for 120 min to minimize the effects of the novel environment on respiratory patterns and parameters, and then monitored in the chamber for at least 4 h. Mice were individually placed into the chamber, and all plethysmography experiments were video‐recorded. Videos of behaviors, plethysmography traces, and breathing parameters were recorded and measured simultaneously. Animal behavioral analysis complied with the video recorded during the lung function test to match the breathing waveforms, which were divided into grooming, scratching, moving, and quiescence. All behavioral analyses were continuous from the test time. Respiratory parameters, including RF (breaths/min), BBI (s) were measured. For LF/HF ratio calculation, defining low‐frequency (LF) RF as < 4 Hz and high‐frequency (HF) RF as ≥ 4 Hz.

The respiratory variability of breathing was assessed through both time domain analysis (CV, SDBB, RMSSD) and nonlinear analysis (SD1, SD2) of the BBI.

Time domain analysis. CV was determined by calculating the ratio of the standard deviation (SD) of the BBI to the mean BBI and expressed as a percentage.

$$CV=Mean_{BBI}/SD_{BBI}\times 100\%$$

SDBB quantifies long‐term variations in respiratory rhythm by calculating the standard deviation of BBI.

$$SDBB=\sqrt{\frac{1}{N-1}\sum_{i=1}^{N}{\left(BBI_{i}-Mean_{BBI}\right)}^{2}}$$

RMSSD shows the square root of the mean of the square of the successive differences between adjacent BBI, quantifies short‐term variability in breathing rhythm.

$$RMSSD=\sqrt{\frac{1}{N-1}\sum_{i=1}^{N-1}{\left(BBI_{i+1}-BBI_{i}\right)}^{2}}$$

Nonlinear analysis. Poincaré plot is a scatter plot of the current BBI plotted against preceding BBI. Poincaré plot analysis is a quantitative visual technique, whereby the shape of the plot is categorized into functional classes. Points above the line of identity indicate BBI that are longer than the preceding BBI, and points below the line of identity indicate a shorter BBI than the previous. Accordingly, the dispersion of point's perpendicular to the line of identity reflects the level of short‑term variability. This dispersion can be quantified by the standard deviation of the distances the points lie from the line of identity. The standard deviation of points along the line of identity reflects the SDBB.

$$\;S{D_{1}}^{2}=\frac{1}{2}\;SDSD^{2}$$

$$\;S{D_{2}}^{2}=2SDSD^{2}\;-\frac{1}{2}SDSD^{2}$$

### 4.5 Virus Injection and Optical Fiber Implantation

The mice were anesthetized with pentobarbital sodium (60 µg g^−1^, i.p.). Additional anesthetics were administered as needed (30% of the original dose, i.p.). The depth of anesthesia was assessed by the lack of corneal and hind‐paw withdrawal reflexes every 30 min. All the surgical procedures were performed under aseptic conditions. Each mouse was placed in a prone position on a stereotaxic device (RWD Life Science, China), and body temperature was maintained at 37°C using a program‐controlled heating pad. The eyes were covered with ophthalmic ointment to prevent drying. Injections were made using a virus‐filled glass pipette (approximately 25 µm tip diameter) connected to a syringe pump (Harvard Apparatus, USA). After exposing the skull and drilling a small hole, the glass micropipette was positioned above the preBötC (bregma: anteroposterior, −6.59 mm; mediolateral, ±1.38 mm; dorsoventral, −5.90 mm), PVT (anteroposterior, −0.80 ∼1.20 mm; mediolateral, 0 mm; dorsoventral, −3.07 mm), CeA (anteroposterior, −0.8 mm; mediolateral, ±2.65 mm; dorsoventral, −4.68 mm), CeL (anteroposterior, −0.95 mm; mediolateral, ±2.83 mm; dorsoventral, −4.68 mm), CeM (anteroposterior, −0.85 mm; mediolateral, ±2.69 mm; dorsoventral, −4.68 mm). The virus was injected unilaterally or bilaterally into the site at 50 nL min^−1^ using a microsyringe pump controller. The pipette was retained for at least 5 min before withdrawal. After that, each mouse received injections of the antibiotic ampicillin (125 mg kg^−1^, i.p.) and analgesic ketorolac (4 mg kg^−1^, i.p.). They were then allowed to recover for four weeks until the resumption of normal activity before the next experimental measurements were taken.

To identify a neural circuit from preBötC to CeA, 80 nL of AAVretro‐hSyn‐Cre and 50 nL of AAV‐hSyn‐hM3Dq‐mCherry were unilaterally injected into the CeA and preBötC of R26‐stop‐EYFP mice. For the relay nucleus neuron counting experiment, 80 nL of AAVretro‐hSyn‐EGFP and 50 nL of AAV‐hSyn‐DIO‐hM3Dq‐mCherry were unilaterally injected into the CeA and preBötC of Vglut2‐Cre mice. For chemogenetic inhibition experiments, 50 nL of AAV‐EF1α‐FDIO‐hM4Di‐EGFP and 80 nL of AAVretro‐EF1α‐DIO‐Flpo were bilaterally injected into the preBötC and PVT of Vglut2‐Cre mice. For optogenetic stimulation, 50 nL of AAV‐EF1α‐DIO‐ChR2‐mCherry or AAV‐Ef1α‐DIO‐mCherry was injected into the preBötC bilaterally. Three weeks after injection, an optical fiber [outer diameter (OD), 200 µm; numerical aperture (NA), 0.22; Sansh Technology] was implanted above the PVT to illuminate the preBötC^Glu^‐PVT terminals. To inhibit the PVT→CeA pathway, 80 nL of AAV‐EF1α‐fDIO‐hM4Di‐EGFP and 80 nL of AAVretro‐DIO‐Flp were stereotaxically injected into the PVT and CeA of Vglut2‐Cre mice, respectively. Concurrently, 50 nL of AAV‐EF1α‐DIO‐ChR2‐mCherry was delivered into the preBötC. Three weeks post‐injection, an optical fiber (OD, 200 µm; NA, 0.22) was implanted above the PVT for subsequent photostimulation. For selective ablation of CeA‐projecting PVT neurons, Vglut2‐Cre mice received bilateral stereotaxic injections of 80 nL AAV‐EF1α‐fDIO‐taCasp3 into the PVT and 80 nL AAVretro‐hSyn‐Flpo‐EGFP into the CeA, with a simultaneous 50 nL injection of AAV‐EF1α‐DIO‐ChR2‐mCherry into the preBötC. After a three‐week recovery period, an identical optical fiber (OD, 200 µm; NA, 0.22) was positioned above the PVT. For fiber photometry, 80 nL of AAVretro‐hSyn‐GCaMP6f and 50 nL of AAV‐hSyn‐DIO‐ChrimsonR‐mCherry or AAV‐hSyn‐DIO‐mCherry were injected into CeA and preBötC. Three weeks after injection, an optical fiber (OD, 200 µm; NA, 0.22) was placed above the PVT. For patch‐clamp experiments, 80 nL of AAVretro‐hSyn‐EGFP and 50 nL of AAV9‐EF1α‐DIO‐ChR2‐mCherry were injected into the CeA and preBötC. To track the axonal distribution of PVT neurons innervated by preBötC, 60nL of AAV1‐hSyn‐Cre and 80 nL of AAV9‐EF1α‐DIO‐EGFP were injected into the preBötC and PVT of C57BL/6J mice. For test the modulation of CeA subdivisions by PVT neurons, 80 nL of AAV1‐hSyn‐Cre and 80 nL of AAVretro‐hSyn‐mCherry were injected into the PVT and CeM. To delineate the electrophysiological connectivity among the PVT, CeL and CeM, 80 nL of AAV‐CaMKIIα‐ChR2‐mCherry and 80 nL of AAV‐hSyn‐EYFP were injected into the PVT, and CeM of C57BL/6J mice. For the assessment of functional connectivity within this tripartite circuit, 80 nL of AAV1‐hSyn‐Cre and 80 nL of either AAV‐EF1α‐DIO‐ChR2‐mCherry or the control virus AAV‐EF1α‐DIO‐mCherry were delivered into the PVT and CeL/CeM of C57BL/6J mice. All viral titers were >10^12^ GC mL^−1^ and were stored in aliquots at −80°C until use.

### 4.6 Behavioral Test

Behavioral testing was performed during the dark phase under red light. Animals were placed in the testing area before testing for at least 2 h for acclimation. The facilities of the behavioral test were cleaned to eliminate residual odors from previous experiments before the test. While each mouse underwent different tests, they were not subjected to the same experiment for less than 1 week. For the optogenetic experiments, an optical fiber fixed in mouse brain was connected to a patch cord, and subsequently the mice were put back into the home cage for at least 10 min, then introduced to the arena for the experiments.

Open field test. The open field arena was a square box (50 × 50 cm) with opaque plexiglass walls within a sound‐attenuated room. Each test mouse was placed in the center of the box and allowed to freely explore its surroundings, which were recorded by a camera attached to a computer. Movement was automatically tracked and analyzed using the Smart v3.0 small animal behavior video recording and analysis system (Version 3.0, Panlab, Spain). The time spent in the center area, the number of entries into the center area, and the total distance traveled were measured.

Elevated plus maze test. A cross‐shaped maze was set up 50 cm above ground. The maze had two 30 cm long opposing closed arms, two 30 cm long opposing open arms, and a 5 cm square in the middle. Test mice were placed in the open arm near the center and allowed to freely explore the maze for 10 min. The number of entries into the open arms, time spent in the open arms, and total distance traveled were calculated using the Smart v3.0.

### 4.7 Fiber Photometry

To record the Ca^2+^ fluorescence of CeA‐projecting PVT neurons by manipulating PVT‐projecting preBötC^Glu^ neurons. Before the experiment, the animals were placed in the experimental field for more than 2 h to adapt to the surrounding environment and to reduce the impact of unfamiliar environments on the experimental results. A single‐channel fiber optic recording system (THINKERTECH, China) was used to adjust the fiber optic output intensity, connect the sCOMS camera and computer, open the data acquisition software, and record the offset value for 5 min. The experimental mouse was gently removed from the cage with its back facing the experimenter. The animal's head recording fiber was connected to the extension cable of the multichannel fiber optic recording system, and the head stimulation fiber was connected to the extension cable of the optogenetic system. The animal was gently placed in the center of the experimental field, and the operator quickly and quietly left, turned on the optogenetic system, and applied 1 Hz, 5 Hz, 10 Hz, 20 Hz, and 589 nm yellow light (10 mW, 20 ms pulse width, 10 s). After the experiment, the experimental arena was cleaned to ensure that the smell left by the animals in the last experiment was eliminated. The values of Ca^2+^ signal changes (ΔF/F) were analyzed by calculating (F−F0)/ F0 (averaged baseline fluorescence signal recorded) using MATLAB.

### 4.8 Optogenetic Stimulation Procedures

The output power of the light at the end of the optical fiber was 10 mW in all experiments, as measured using an optical power meter (PM20; Thorlabs, USA). The blue (473 nm) or yellow (580 nm) light was controlled using an LED source stimulator (Newdoon Inc., China). For behavioral tests, we connected the fiber implanted in mice with a patch cord of a laser stimulator and placed the mice into an open field or plus maze. The laser controller was then opened with blue light (473 nm, 10 mW, 20 ms pulse width, 10 Hz, 10 min), and the locomotor activity of the mice was recorded for 10 min.

For 40 min long‐term respiration measurements, the optical fiber was connected to the patch cord of the laser stimulator, and the free‐behaving mice were irradiated with 473 nm blue light (10 mW, 20 ms pulse width, 10 Hz, 40 min). Respiratory parameters were monitored for 40 min using WBP.

For fiber photometry, light stimulation was applied at frequencies of 1, 5, 10, and 20 Hz, along with 589 nm yellow light (10 mW, 20 ms pulse width, 10 s).

For stimulation of target neurons in superfused brain slices, the optical fiber was connected to the patch cord of the laser stimulator. The PVT brain slices were irradiated with 473 nm blue light (10 mW, 20 ms pulse width, 5 Hz, 10 s). The CeA brain slices were irradiated with a single exposure of 473 nm blue light (10 mW, 10 ms pulse width).

### 4.9 Chemogenetics

To chemogenetically activate or inhibit the specific neurons, four weeks after injections of the virus encoding hM3Dq or hM4Di into target regions, mice were intraperitoneally injected with saline or CNO (2 mg kg^−1^, dissolved in 0.9% saline) and placed back into their home cage. Thirty minutes after drug administration, the mice were subjected to the corresponding operations.

### 4.10 Immunohistochemistry

The animals were deeply anesthetized with urethane (1.8 g kg^−1^, i.p.) and transcardially perfused with chilled saline followed by paraformaldehyde (PFA, 4% in PBS, pH 7.4). After decapitation, the brains were removed and post‐fixed for 24 h in 4% PFA at 4°C and subsequently transferred to a 30% PBS‐buffered sucrose solution until saturation (24–36 h). Tissues were embedded in OCT compound and stored at −80°C before use. Coronal sections were cut at 25 µm thickness using a freezing microtome (CM1950; Leica Microsystems, Germany). Sections were blocked with 5% bovine serum albumin (BSA) in PBS (0.25% Triton X‐100 in PBS) for 30 min at room temperature (23–24°C), followed by incubation with primary antibodies in 2% BSA‐PBS overnight at 4°C. The sections were washed with PBS (3 × 5 min) and incubated with fluorescent secondary antibodies at room temperature for 1 h. All rinses and incubations were performed on a shaker at low speed. After rinsing with PBS (3 × 5 min), the sections were mounted on slides using Vectashield Antifade Mounting Medium (Vector Laboratories, Burlingame, CA, USA) for visualization. Images of whole‐brain sections were captured using a laser‐scanning confocal microscope (LSM 800, Carl Zeiss, Germany) and processed using the ZEN software (Zeiss, Germany).

The primary antibodies used were as follows: chicken anti‐GFP (dilution 1:2000, Cat # ab13970, RRID: AB_300798, Abcam), rabbit anti‐mCherry (dilution 1:1000, Cat # NBP2‐25157, RRID: AB_2753204, Novus Biologicals), guinea pig anti‐c‐Fos (dilution 1:1000, Cat # 226308, RRID: AB_2905595, Synaptic Systems). The fluorophore‐conjugated secondary antibodies used were: goat anti‐chicken IgY H&L (Alexa Fluor 488) (dilution 1:1000, Cat # ab150169, RRID: AB_2636803, Abcam), donkey anti‐rabbit IgG H&L (Alexa Fluor 555) (dilution 1:1000, Cat # ab150074, RRID: AB_2636997, Abcam), goat anti‐guinea pig IgG H&L (Alexa Fluor 647) (dilution 1:500, Cat # ab150187, RRID: AB_2827756, Abcam).

### 4.11 RNAscope Fluorescence In Situ Hybridization

To detect the neural type of the PVT, FISH was conducted using the RNAscope Multiplex Fluorescent Reagent Kit V2 (ACDbio) according to the manufacturer's instructions. In short, fresh‐frozen brains from adult male C57BL/6 J mice were fixed with paraformaldehyde (PFA, 4%, 4°C) after anaesthetization, and brain tissue was collected and immersed in PFA (4°C) for 24 h. Subsequently, the tissue was dehydrated through a gradient of 15% and 30% sucrose phosphate buffer solution (PBS) and embedded in OCT with storing at −80°C. Coronal sections at a thickness of 18 µm were obtained using a cryostat (CM1950, Leica Microsystems, Wetzlar, Germany). *Slc17a6*, *Slc32a1* mRNA signal was labeled by using the RNAscope multichannel second‐generation fluorescence kit (Advanced Cell Diagnostics), according to the manufacturer's instructions. Sections were cover slipped using Diamond Prolong antifade mounting medium with DAPI (ThermoFisher Scientific).

### 4.12 Electrophysiology

Transverse brain slices of mice were prepared after rapid decapitation under anesthesia (5% pentobarbital sodium, 1.5 mL kg^−1^, i.p.). The mesocephalon was dissected, and coronal slices (250 µm) were cut on a vibrating microtome (VT1200S, Leica Biosystems, Germany) in ice‐cold sucrose‐containing solution (in mM: 260 sucrose, 3 KCl, 5 MgCl_2_, 1 CaCl_2_, 1.25 NaH_2_PO_4_, 26 NaHCO_3_, 10 glucose, and 1 kynurenic acid). Slices were incubated for 30 min to 1 h at 37°C and subsequently at room temperature in normal Ringer's solution containing the following components (in mM): 130 NaCl, 3 KCl, 2 MgCl_2_, 2 CaCl_2_, 1.25 NaH_2_PO_4_, 26 NaHCO_3_, and 10 glucoses. All the cutting and incubation solutions were bubbled with 95% O_2_ and 5% CO_2_. We targeted EYFP‐expressing PVT neurons for patch‐clamp recordings from coronal slices in chambers using fixed‐stage fluorescence microscopes equipped with infrared Nomarski optics (Carl Zeiss AxioExaminer or Olympus Optical BX51WI). For recording, slices were perfused with oxygenated aCSF (in mM: 124 NaCl, 3 KCl, 1.2 NaH_2_PO_4_, 1.2 MgSO_4_, 25 NaHCO_3_, 11 D‐glucose, 0.4 L‐ascorbic acid and 2 CaCl_2_, saturated with 95% O_2_%–5%CO_2_, pH 7.4, 300 mOsm) at 31°C. Recordings were performed in a whole‐cell configuration. Signals were amplified using a Multiclamp 700 B amplifier (Molecular Devices), filtered at 1 KHz (Bessel low‐pass), and sampled at 10 KHz (Digidata 1440A, Molecular Devices). Recording electrodes (4–6 MΩ) were filled with a solution containing the following components (in mM): 10 NaCl, 130 K^+^ gluconate, 11 EGTA, 1 CaCl_2_, 10 HEPES, 1 MgCl_2_, 2 MgATP, and 0.2 NaGTP, pH 7.3 (295–300 mOsm).

Whole‐cell patch clamp recordings were then made to assess monosynaptic or polysynaptic connections. Cells with a resting membrane potential less than–45 mV upon initial membrane rupture were not considered for further analysis. Cells were clamped at –60 mV for EPSCs and at 0 mV for IPSCs. No leak subtractions, liquid junction potential corrections, or series resistance compensations were performed. Data were analyzed off‐line using either Clampfit (Molecular Devices) or Spike2 software (Cambridge Electronic Design). EPSCs or IPSCs were evoked by photostimulation of ChR2‐expressing neurons. To test whether the postsynaptic currents recorded in PVT neurons were elicited by direct synaptic connections, TTX (1 µM) and 4‐AP (100 µM) were added into aCSF for perfusion. To test whether the recorded EPSCs were mediated by glutamate receptors, CNQX (10 µM) was bath‐applied. Whole‐cell recordings from CeM and CeL neurons were performed using the same protocol.

### 4.13 Human Study Design

First, to investigate the impact of a slow breathing pattern on human anxiety, we assessed the anxiety levels of healthy volunteers using the Beck Anxiety Inventory (BAI) questionnaire before and after trial. Subjects were randomly divided into two trials (eupnea breathing trial and slow breathing trial). Each trial consisted of three 5‐min phases, with the first and third phases requiring participants to keep rest with their eyes closed. In the second phase, the eupnea breathing trial required participants to maintain natural breathing with their eyes closed, and the slow breathing trial required participants to take slow and deep breaths with their eyes closed. For the participants to master the essentials of slow breathing, a 1–5 min training session was conducted before the start of the experiment to slow down the respiratory rate and master the rhythm of breathing. The subjects were reminded to pay attention to the rhythm of breathing when they started the slow breathing trial.

Second, we conducted the same experiment after scoring the BAI of epilepsy patients and simultaneously recorded intracranial electrophysiological signals. Patients were subjected to two trials with a time interval of at least 3 h, and the order of the trials was randomized. The whole experiment was synchronized with the recording of brain electrophysiological activity through intracranial electrodes.

### 4.14 Evaluating the Effect of Slow Breath on Neural Activity of the Amygdala

Data acquisition, signal preprocessing, and activity level analysis for oscillations as described before [65]. The experiment was started at least 3 days after the implantation of SEEG electrodes to minimize the effects of general anesthesia on the brain and body. In this study we focused only on the effect of slow breathing on amygdala activity, so subjects whose epileptic foci were identified as amygdala or whose amygdala epileptiform discharges were too frequent were excluded. All participants were seizure‐free for at least 12 h before the experiment and no participants experienced seizures during the experiment. Electrode implantation location was determined by co‐registering preoperative MRI and postoperative CT. Intracranial electrodes were reconstructed to precisely outline the tip and trajectory of each electrode shaft, and the location of each contact was calculated based on the length and spacing of the electrode contacts. The electrode used in this study contains 8–20 contacts with a diameter of 0.8 mm at a spacing of 2 mm. In order to determine which contacts were in the amygdala, the reconstruction was overlaid onto ASEG atlases.

The absolute power spectral analysis (absPSD) of each epoch was calculated using the windowed fast Fourier transform with a 1 s sliding window, a 0.5 s overlap, and an NFFT of 2048. These parameters provide 1025 frequency points within the 0–500 Hz range at a frequency resolution of 0.488 Hz. The power of a specific frequency band oscillation was defined as the area under the PSD curve within its frequency range. There were six oscillations: delta (δ, 1–4 Hz), theta (θ, 4–8 Hz), alpha (α, 8–12 Hz), beta (β, 12–30 Hz), low‐gamma (lγ, 30–60 Hz), and high‐gamma (hγ, 60–90 Hz). For each bipolar signal, the power of oscillation of all epochs was averaged.

### 4.15 Statistical Analysis

All experiments and data analyses were performed in a blinded manner, including breathing, behavioral, immunohistochemical, and electrophysiological analyses. Data were analyzed using GraphPad Prism v.9.4.0, Smart V3.0, ZEN, Spike2, Adobe Illustrator 2022, Clampfit, Microsoft Office 2013, and MATLAB R2017a. For representative photomicrographs, each experiment was repeated in at least three mice with multiple brain slices, with consistent results. The experimental and control animals were randomly assigned throughout the study. Two‐group unpaired comparisons with one factor were analyzed using a two‐tailed unpaired *t‐*test for data with a normal distribution or two‐tailed Mann‐Whitney test for data without a normal distribution; two‐tailed unpaired *t‐*test with Welch's correction was used for data with a normal distribution but heterogeneous variance. Two‐group paired comparisons with one factor were analyzed using two‐tailed paired *t* test for data with a normal distribution or two‐tailed Wilcoxon matched‐pairs signed rank test for data without a normal distribution. Three or more groups of unpaired comparisons with one factor were analyzed using ordinary one‐way ANOVA for data with a normal distribution, with post‐hoc Dunnett's, Tukey's, or Bonferroni's multiple comparison tests. For data without a normal distribution, Brown‐Forsythe and Welch ANOVA tests with Šídák's multiple comparisons test or Kruskal‐Wallis test were used with post‐hoc Dunn's multiple comparisons test. Three‐group paired comparisons with one factor were analyzed using repeated measures one‐way ANOVA for data with a normal distribution, with post‐hoc Tukey's multiple comparison test. For data without a normal distribution, the two‐sided Friedman test was used with post‐hoc Dunn's multiple comparisons test. Comparisons between groups with two factors were analyzed using two‐way ANOVA, with Bonferroni's, Šídák's or Tukey's multiple comparisons test. Two‐way ANOVA followed by uncorrected Fisher's LSD was used to compare the two groups. The Spearman test was applied to evaluate each correlation since most data did not conform to the normal distribution. Statistical details, including F‐values, t‐statistics, exact *p*‐values, and statistical test types, are described in the supporting information (Table S1). All data are shown as mean ± SEM with statistical significance set as ^*^ *p* < 0.05, ^**^ *p* < 0.01, ^***^ *p* < 0.001, ^****^ *p* < 0.0001.

## Author Contributions

S.B. and X.W. designed the experiments, analyzed the data, and wrote the manuscript. H.L. carried out the evaluation of the effect of slow breathing on neural activity in the amygdala experiments and analyzed the data. Z.Y., Y.L., and X.C. performed breathing measurements and analyzed the WBP results. T.D. and L.S. conducted ex vivo electrophysiological recording. J.Z. performed behavior tests. N.M. analyzed immunohistochemical data. L.M., J.D., J.X. conducted the assessment of epilepsy patients, performed electrode implantation, and collected SEEG data. T.‐F.Y., S.W., and F.Yuan were responsible for the study concept and design. S.W. and F.Y. also obtained research funding. All authors approved the version to be published.

## Conflicts of Interest

The authors declare no conflicts of interest.

## Supporting information




**Supporting File 1**: advs74469‐sup‐0001‐SuppMat.pdf.

**Supporting File 2**: advs74469‐sup‐0002‐TableS1.xlsx.

## Data Availability

The data that support the findings of this study are available from the corresponding author upon reasonable request.
