# Supplementary material for: Respiratory‐Limbic Coupling via a Thalamic Circuit Alleviates Anxiety
Source: Adv Sci (Weinh). 2026 Mar 2;13(24):e17477. doi: 10.1002/advs.202517477 (PMC13115990; doi:10.1002/advs.202517477)
Supplement: Supplementary file 1 — Supporting File 1: advs74469‐sup‐0001‐SuppMat.pdf. [file ADVS-13-e17477-s002.pdf]

Supporting Information

Respiratory-limbic coupling via a thalamic circuit alleviates anxiety

Shangyu Bi, Xiaoyi Wang, Huichun Luo, Ziteng Yue, Tianjiao Deng, Yuhang Liu, Xinxin Chen, Jianxu Zhao, Luo Shi, Ning Ma, Lingyan Mao, Jing Ding, Jiwen Xu, Ti-Fei Yuan, Sheng Wang* and Fang Yuan**

Supporting information include:

Supplementary Figure 1-12

Supplementary Table 1

Figure S1

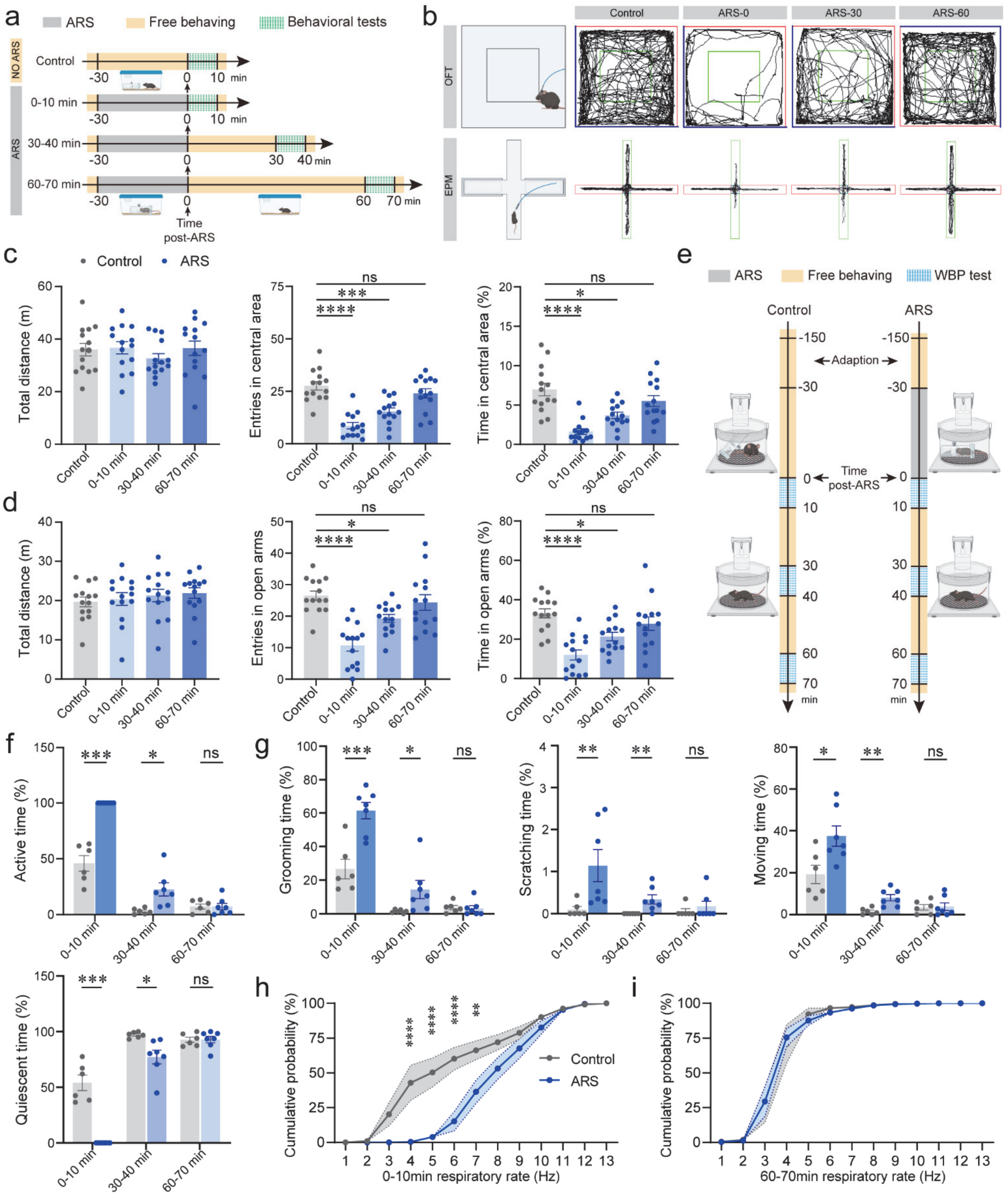

Figure S1 ARS induces behavioral and respiratory changes, related to Fig. 1. a) Experimental timeline for ARS and subsequent phenotyping. b) Schematic and representative trajectories of the OFT

(top) and EPM (bottom). c, d) ARS-treated mice exhibited anxiety-like phenotypes, with significantly fewer entries and less time spent in the center of the OFT (c) and the open arms of the EPM (d) at 0–10 min and 30–40 min post-stress, normalizing by 60–70 min (n = 14 mice per group). e) Schematic of the WBP setup. f) Cumulative time spent in active and quiescent states post-ARS (n = 6 mice for control, n = 7 mice for ARS). g) Cumulative time spent in specific active behaviors (n = 6 control, n = 7 ARS). h, i) Cumulative distribution of RF at 0–10 min (h) and 60–70 min (i) post-ARS (n = 7 mice per group). Statistical significance: All data are presented as the mean ± SEM. $^{*}p < 0.05$, $^{**}p < 0.01$, $^{***}p < 0.001$, $^{****}p < 0.0001$, determined by one-way ANOVA with Tukey's multiple comparisons test (c, d), two-way ANOVA with Bonferroni's multiple comparisons tests (h, i) and two-tailed unpaired *t* test (f, g).

Figure S2

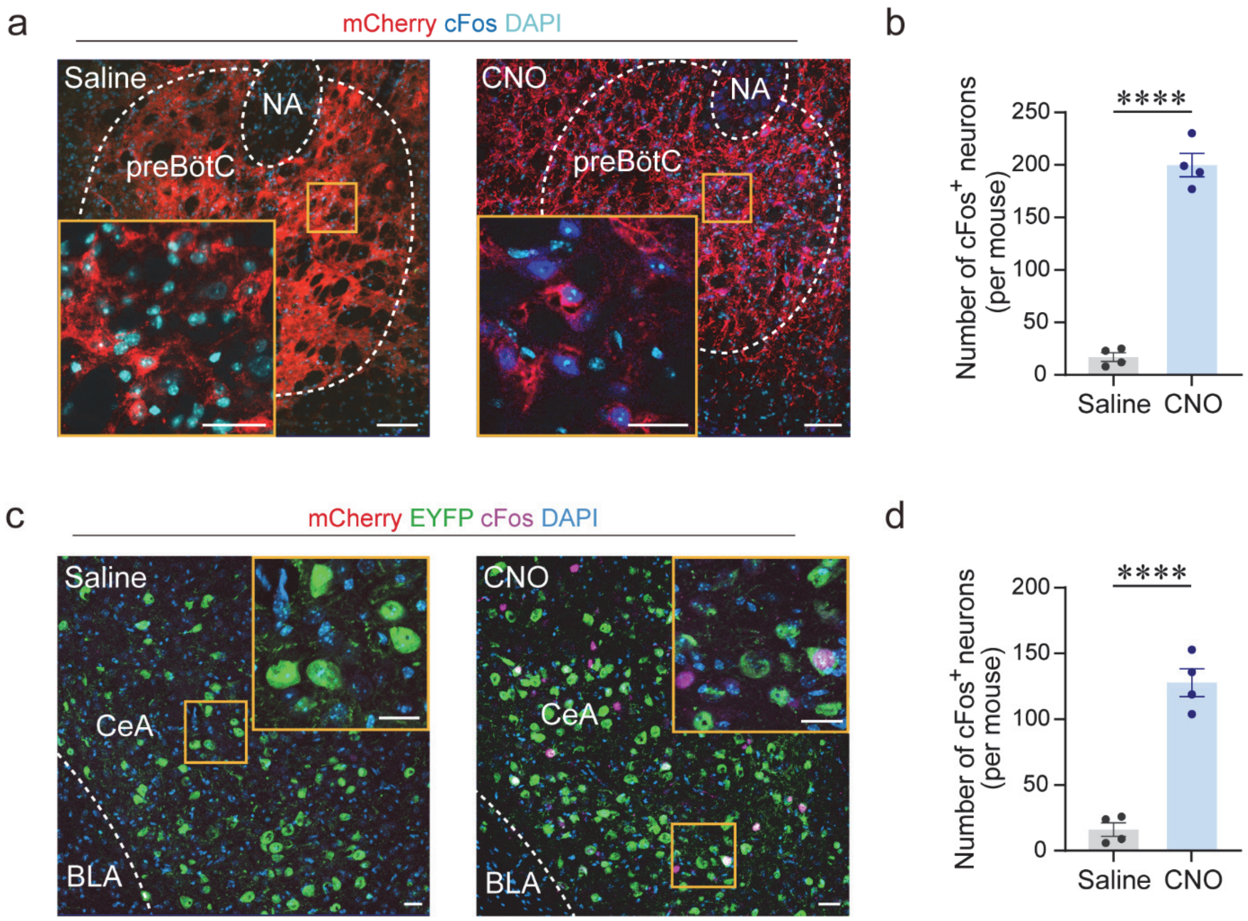

Figure S2 Immunohistochemical validation of chemogenetic stimulation, related to Figure 2.

a) Representative immunostaining for mCherry (virus expression), cFos (neuronal activation) and DAPI in the preBötC following saline (left) or CNO (right) administration. Insets show higher magnification of the boxed areas. Scale bars, 50 µm. b) Quantification of $cFos^{+}$ neurons in the preBötC, confirming significant activation by CNO. Counts were performed on four equally spaced coronal sections per mouse (bregma: –7.10 to –6.80 mm, 25 µm thickness; 75 µm interval) from n = 4 mice per group. c) Representative images showing $EYFP^{+}$ neurons and $cFos^{+}$ (pink) and DAPI in the CeA after saline (left) or CNO (right) treatment. Insets are magnified views of the indicated areas. Scale bars, 50 µm. d) Quantification of $cFos^{+}$ neurons in the CeA of the same animals, using 13 equally spaced sections (bregma: –1.90 to –0.70 mm; 25 µm thickness; 75 µm interval) from n = 4 mice per group. Statistical significance:

$^{****}p < 0.0001$ by two-tailed unpaired t test (b, d). Abbreviations: NA, nucleus ambiguous; BLA, basolateral amygdaloid nucleus, anterior; CeA, central amygdala.

Figure S3

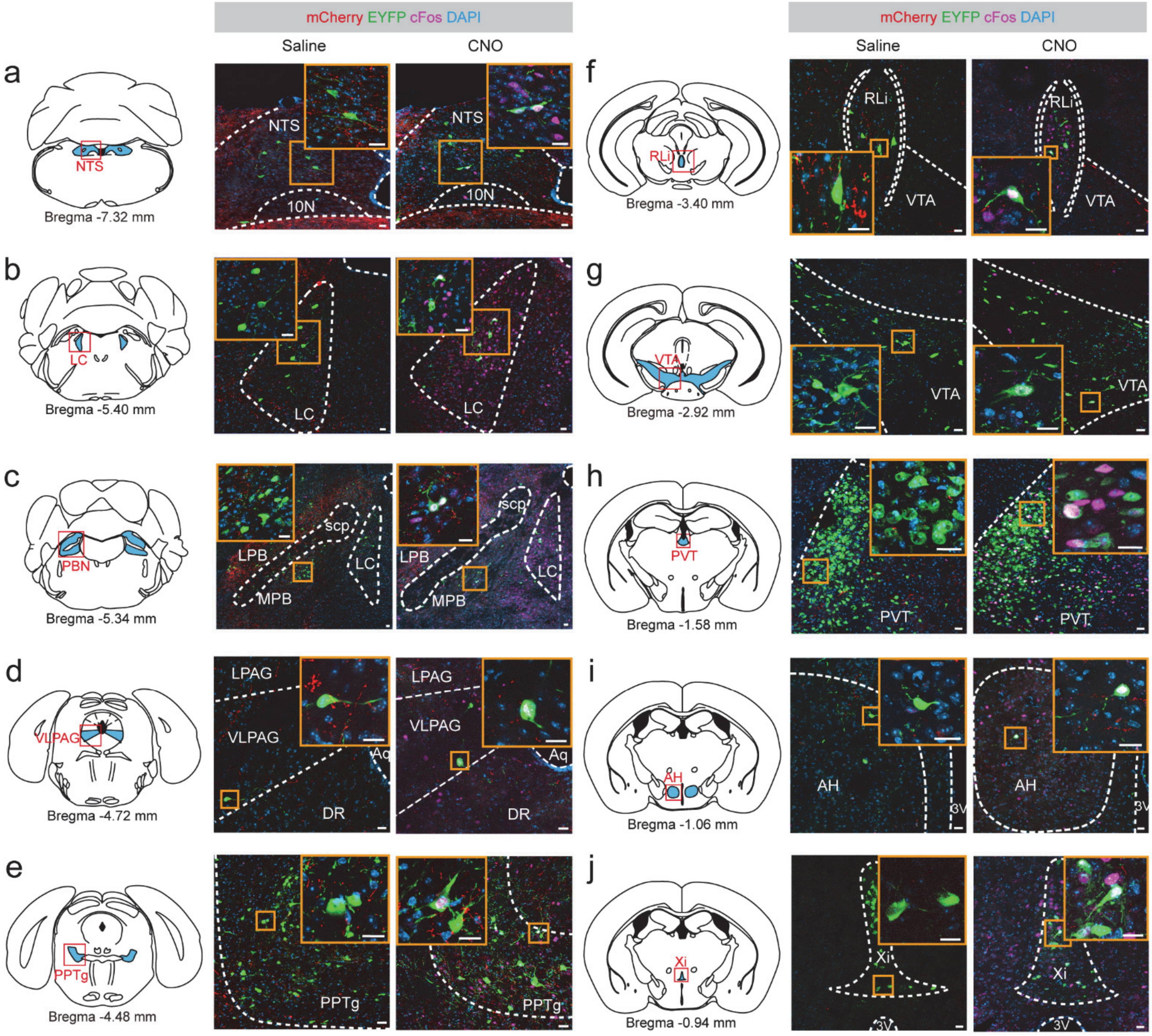

Figure S3 Whole-brain mapping of preBötC→CeA relay neurons, related to Figure 2.

Chemogenetic activation of preBötC neurons in R26-stop-EYFP mice (n = 3 per group), followed by co-localization analysis of $EYFP^+$ (CeA-projecting) and $cFos^+$ (activated) neurons, identified potential relay nuclei connecting the preBötC to the CeA. Representative images (saline, left; CNO, right) show double-labeled neurons distributed in the NTS (a), LC (b), PBN (c), VLPAG (d), PPTg (e), RLi (f), VTA (g), PVT (h), AH (i), Xi (j). Left: Saline, right: CNO. Scale bars, 20 μm (low resolution images and inserts).

Abbreviations: 3V, 3rd ventricle; 10N, vagus nerve nucleus; AH, anterior hypothalamic area; Aq, aqueduct; DR, dorsal raphe nucleus; LC, locus coeruleus; LPBN, lateral parabrachial nucleus; LPAG, lateral periaqueductal gray; MPBN, medial parabrachial nucleus; NTS, nucleus tractus solitarius; PBN, parabrachial nucleus; PVT, paraventricular thalamic nucleus; RLi, rostral linear nucleus; PPTg, pedunculo-pontinetegmental nucleus; scp, superior cerebellar peduncle. vlPAG, ventrolateral periaqueductal gray; VTA, ventral tegmental area; Xi, xiphoid thalamic nucleus.

Figure S4

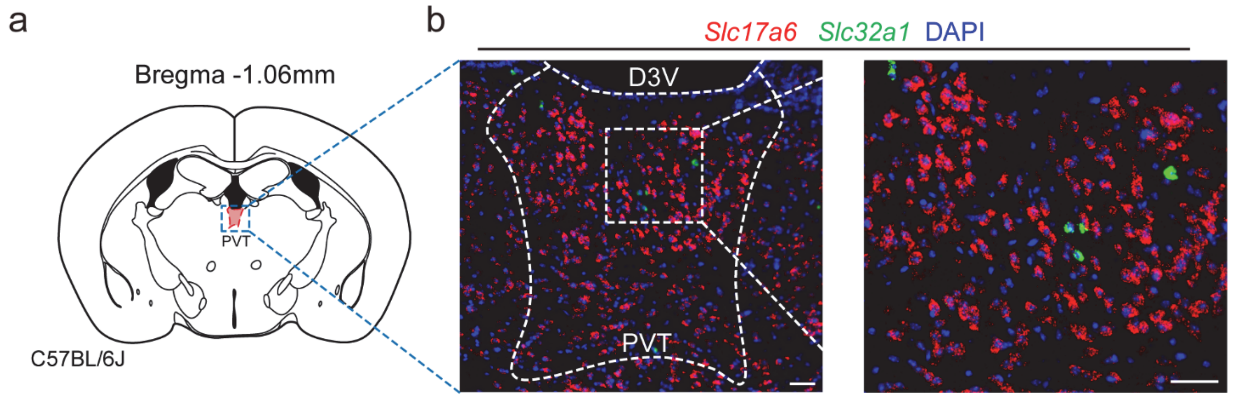

Figure S4 Neurochemical characterization of PVT neurons, related to Figure 2.

a) Schematic of a coronal section indicating the location of the PVT. b) RNAscope *in situ* hybridization for *Slc17a6* (Vglut2, red; glutamatergic) and *Slc32a1* (Vgat, green; GABAergic) transcripts in the PVT. The left panel shows a low-magnification view; the right panel is a magnified image of the boxed region. The majority of PVT neurons express the glutamatergic marker, consistent with a predominantly excitatory phenotype. Scale bars: 50 µm.

Figure S5

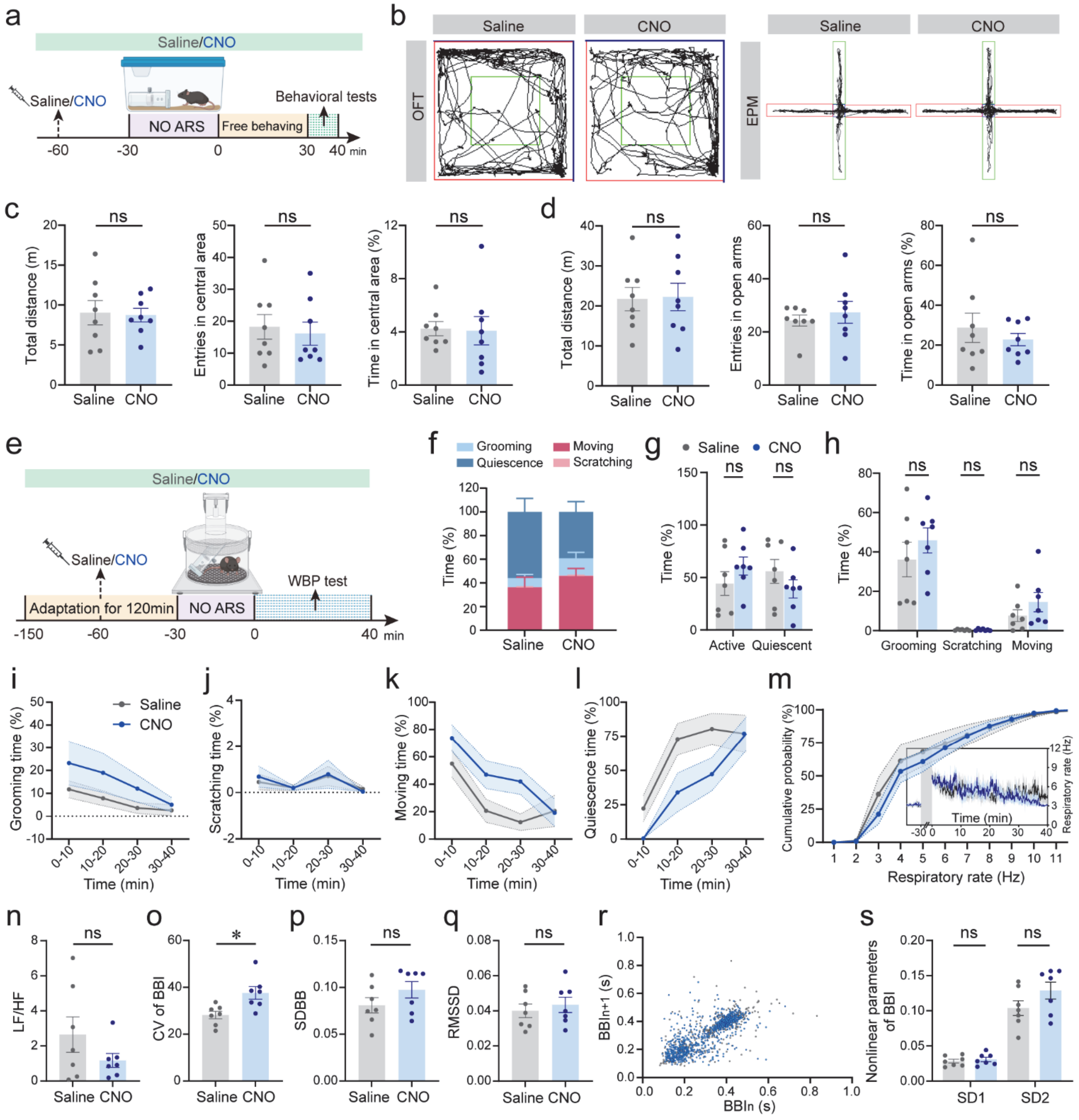

Figure S5 Inhibition of the preBötC^{Glu} → PVT pathway does not alter baseline behavior or respiration, related to Figure 3.

a) Schematic of the chemogenetic inhibition experiment in unrestrained (non-stressed) mice. b) Representative trajectories of the OFT (left) and EPM (right) tests in unrestrained mice following CNO or saline administration. c, d) Quantitative analysis confirmed no significant effect of pathway inhibition on OFT (c) or EPM (d) performance (n = 8 mice per group). e) Timeline for respiratory recordings. f) Percentage of time spent in grooming, scratching, moving, and quiescence during a 40-min session (n = 7 per group). g) Cumulative time in active *vs.* quiescent states. h) Cumulative duration of each active behavior. i-l) Time-course of grooming (i), scratching (j), moving (k), and quiescence (l) in a 10-min interval across a 40-min period (n = 7 per group). m) Cumulative distribution plot of RF. Insert: time-course traces of RF. n) Ratio of low-frequency to high-frequency breathing (LF/HF). o-q) Quantitative analyses of CV (o), SDBB (p), RMSSD (q) of BBI (n = 7 per group). r) Poincaré plot of BBI. s) Nonlinear variability parameters (SD1, SD2) from Poincaré analysis (n = 7 per group). Statistical significance: $^{*}p < 0.05$. Statistical analyses were performed using two-way ANOVA with Bonferroni's multiple comparisons tests (i-m) and two-tailed unpaired *t* test (c, d, g, h, n-q, s). All data are presented as the mean ± SEM.

Figure S6

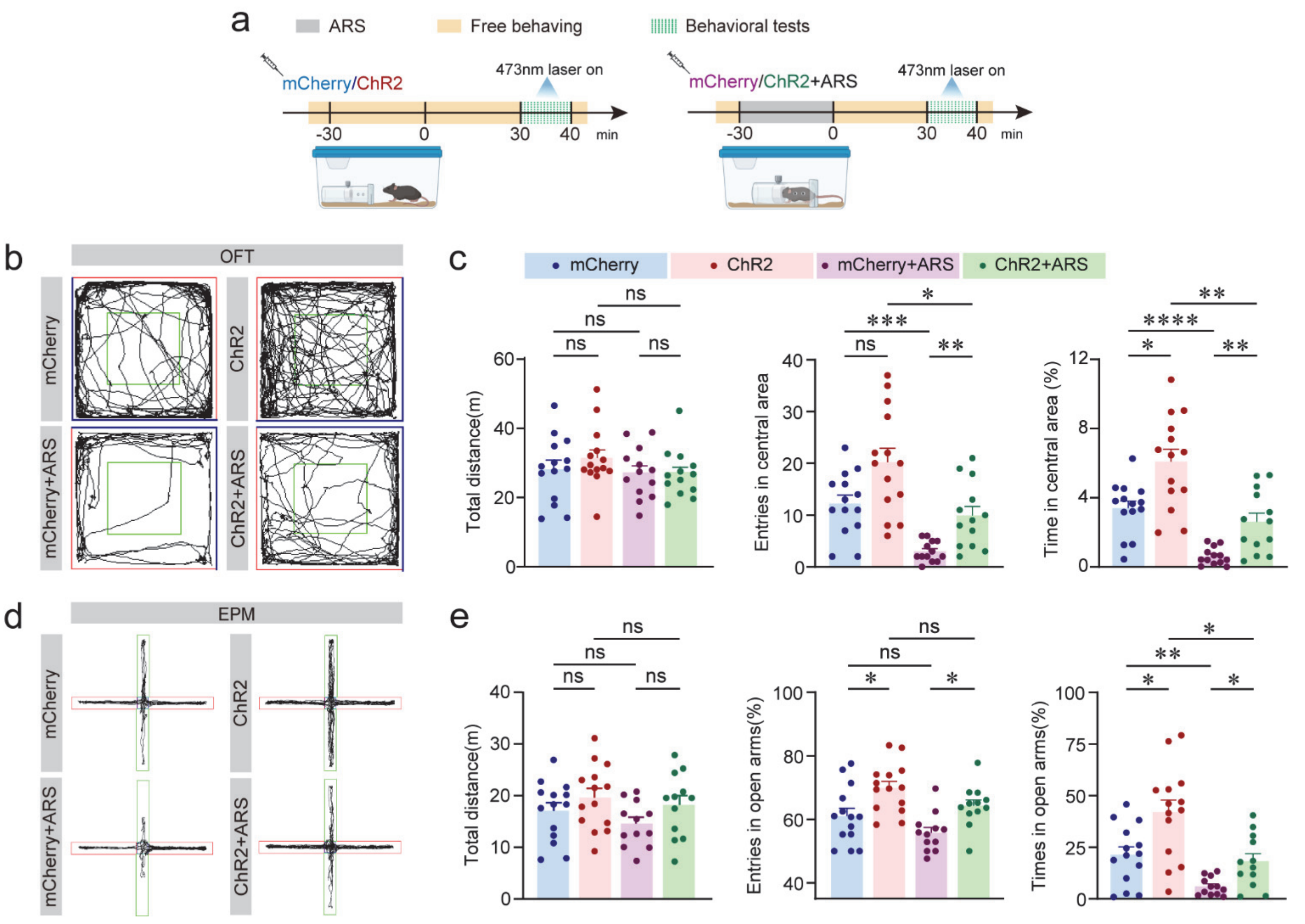

Figure S6 Photostimulation of preBötC^{Glu} neurons projecting to the PVT alleviates ARS-induced anxiety-like phenotypes, related to Figure 4.

a) Experimental timeline for optogenetic stimulation and behavioral testing. b) Schematic of representative trajectories of the OFT. c) Quantification of anxiety-like behavior in the OFT under baseline and ARS conditions (n = 14 for both mCherry and ChR2, n = 13 for both mCherry + ARS and ChR2 + ARS). d) Schematic of representative trajectories of the EPM. e) Quantification of EPM performance across the same groups (n = 14 for mCherry and ChR2, n = 12 for mCherry + ARS and ChR2 + ARS). Statistical significance: All data are presented as the mean ± SEM. $^{*}p < 0.05$, $^{**}p < 0.01$, $^{***}p < 0.001$, $^{****}p < 0.0001$, determined by one-way ANOVA with Bonferroni's multiple comparisons test (c, e).

Figure S7

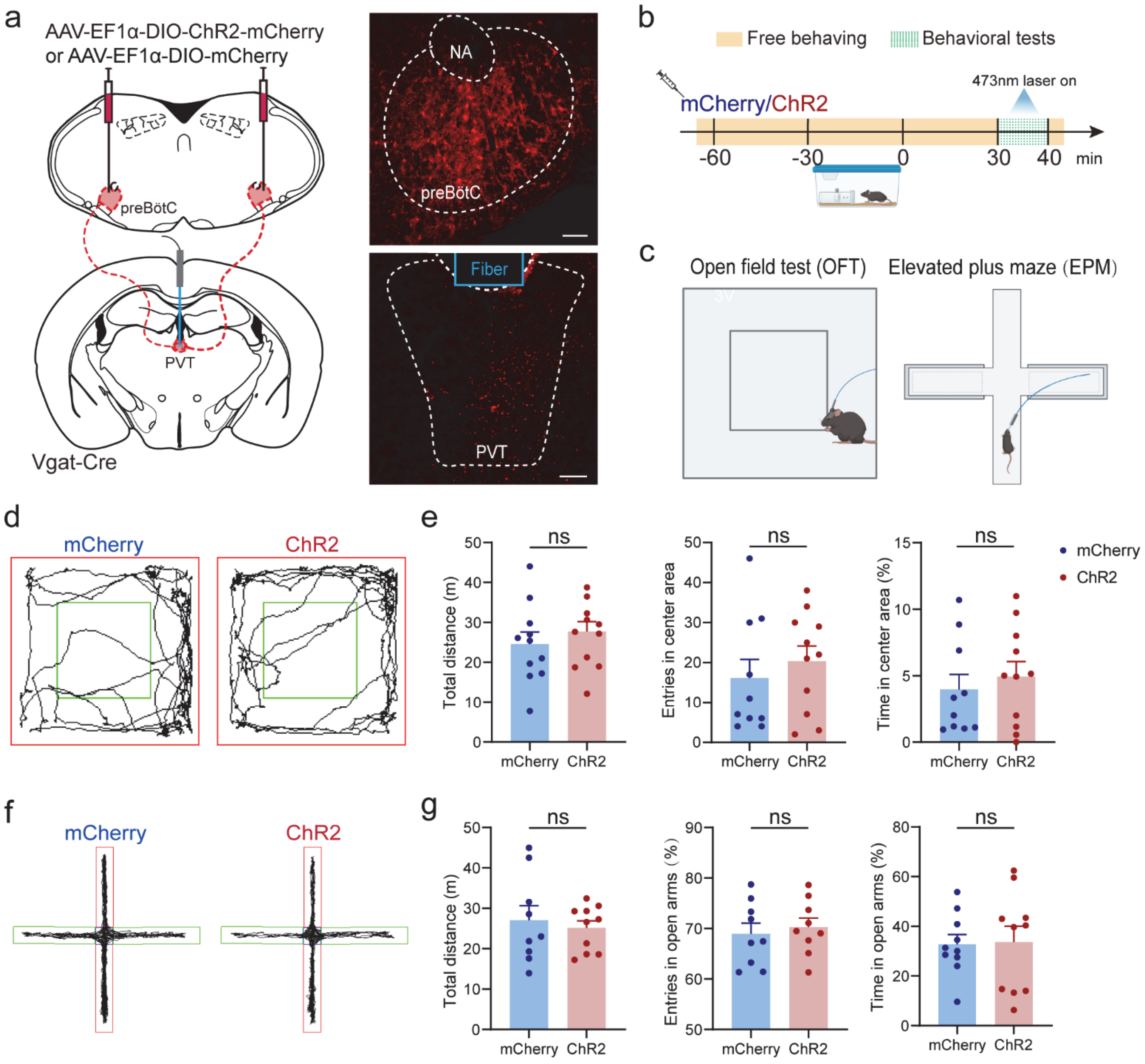

Figure S7 Stimulation of preBötC^{GABA}→PVT projections does not affect baseline anxiety-like phenotypes, related to Figure 4.

a) Left: Optogenetic strategy for targeting GABAergic preBötC neurons in Vgat-Cre mice. Right: Confirmation of ChR2-mCherry expression in the preBötC and corresponding axonal projections within the PVT. Scale bars, 100 µm. b) Schematic of the experimental procedure in unrestrained mice. c)

Integration of behavioral testing with optical stimulation. d) Representative traces of locomotor trajectories in the OFT. e) Quantification of OFT performance showed no significant effect of GABAergic pathway stimulation (n = 10 for mCherry and n = 11 for ChR2). f) Representative traces of locomotor trajectories in the EPM. g) Quantification of EPM performance similarly showed no significant difference (n = 10 for mCherry and n = 11 for ChR2). Statistical analyses were conducted using two-tailed unpaired *t* test (e, g). All data are presented as the mean ± SEM.

Figure S8

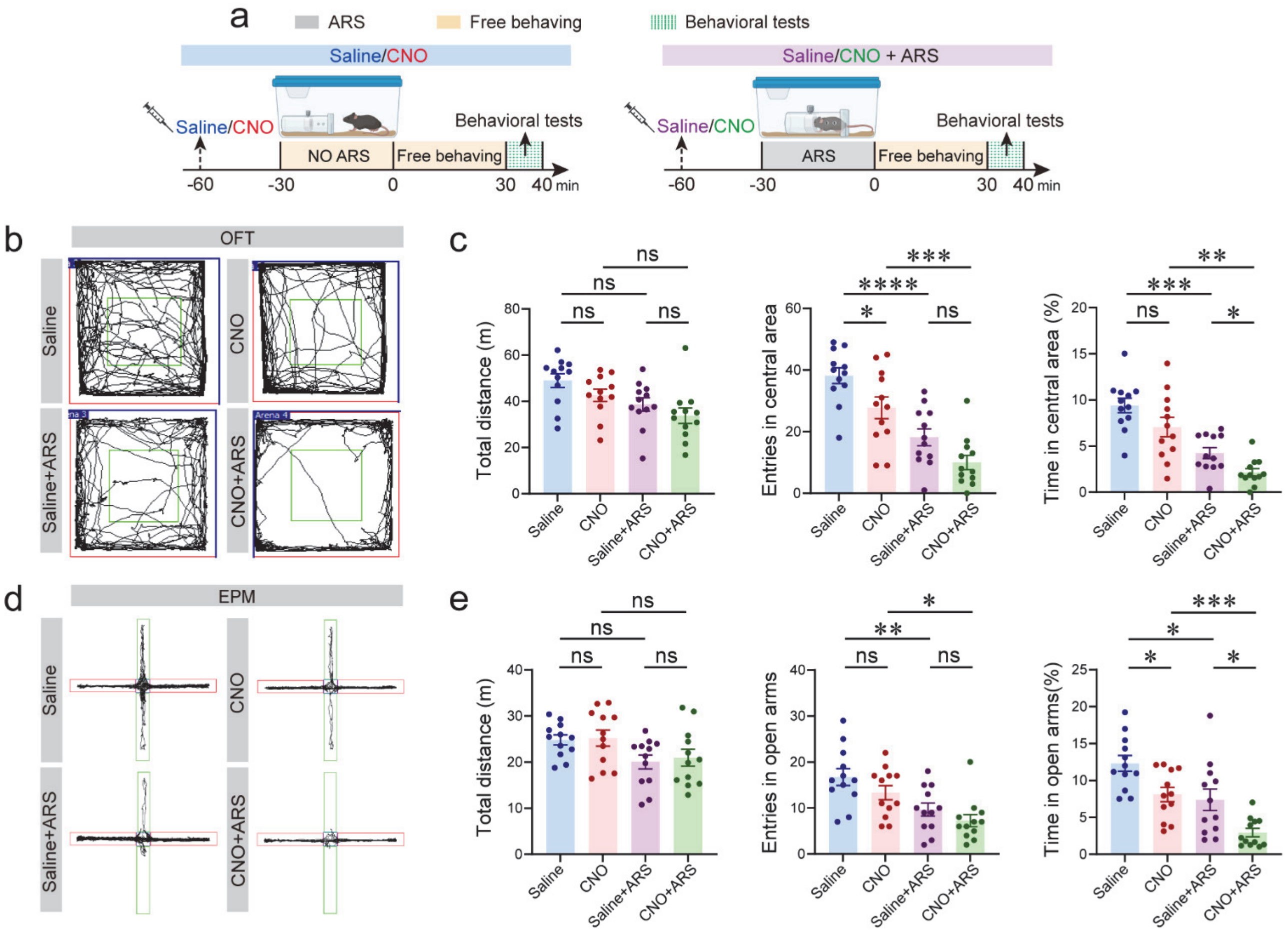

Figure S8 Inhibition of the PVT→CeA pathway eliminates the anxiolytic effect of preBötC^{Glu}→PVT stimulation, related to Figure 5.

a) Schematic of experimental procedure. b) Schematic and representative trajectories of the OFT. c) Quantification of OFT performance in unrestrained and ARS-treated mice across all stimulation/inhibition groups (n = 12 mice per group). d) Schematic and representative trajectories of the EPM. e) Quantification of EPM performance for the same groups (n = 12 for each group). Significance levels: All data are presented as the mean ± SEM. $^{*}p < 0.05$, $^{**}p < 0.01$, $^{***}p < 0.001$, $^{****}p < 0.0001$. Statistical analyses were performed using one-way ANOVA with Tukey's multiple comparisons test (c, e).

Figure S9

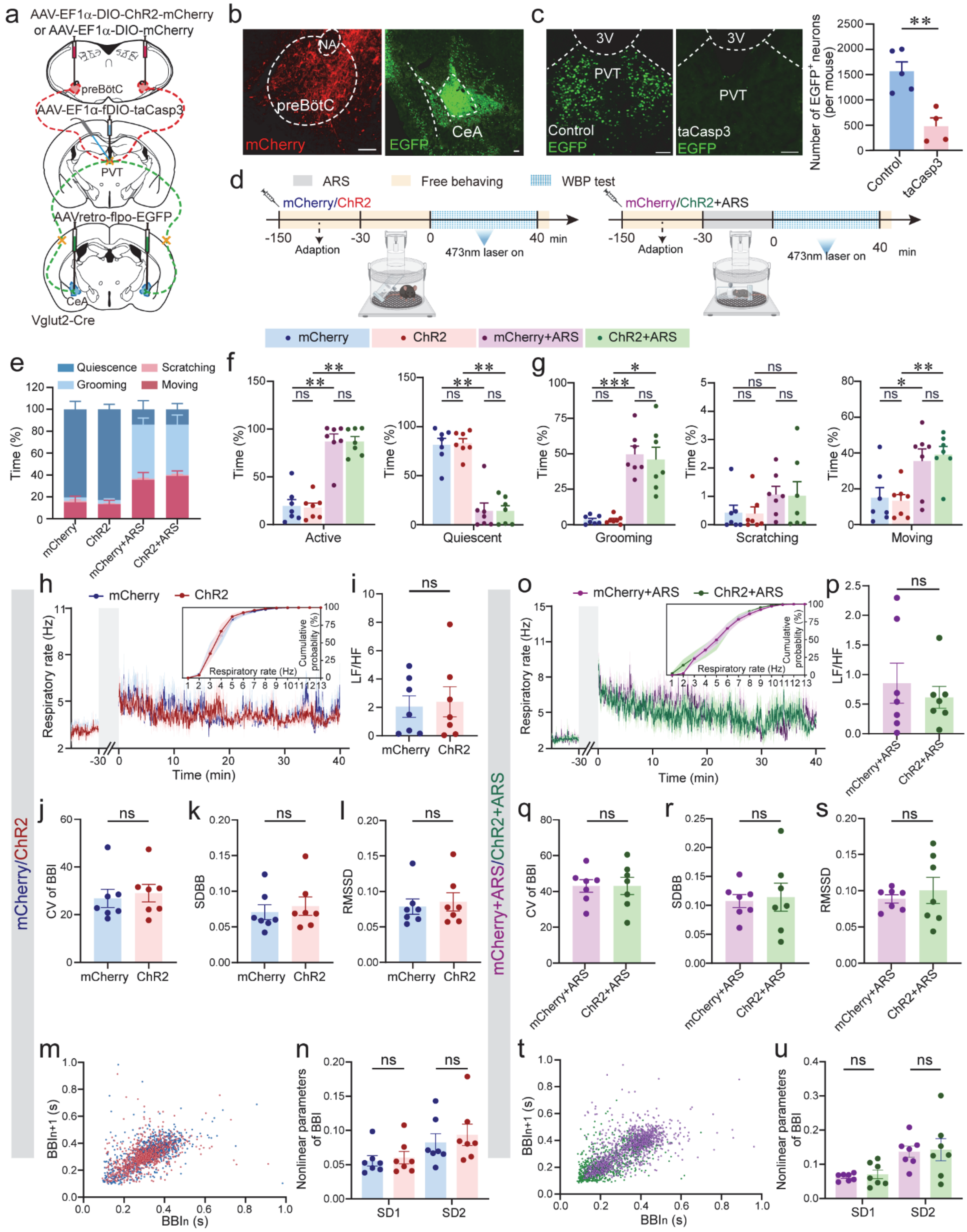
a
AAV-EF1α-DIO-ChR2-mCherry
or AAV-EF1α-DIO-mCherry
preBötC
AAV-EF1α-fDIO-taCasp3
PVT
AAVretro-flpo-EGFP
CeA
Vglut2-Cre
b
NA
preBötC
mCherry
EGFP
CeA
c
3V
PVT
Control
EGFP
taCasp3
Number of EGFP+ neurons (per mouse)
Control
taCasp3
d
ARS
Free behaving
WBP test
mCherry/ChR2
mCherry/ChR2+ARS
Adaption
473nm laser on
min
mCherry
ChR2
mCherry+ARS
ChR2+ARS
e
Quiescence
Scratching
Grooming
Moving
Time (%)
f
Active
Quiescent
g
Grooming
Scratching
Moving
h
Respiratory rate (Hz)
Time (min)
Cumulative probability (%)
Respiratory rate (Hz)
i
LF/HF
o
p
j
CV of BBI
k
SDBB
l
RMSSD
q
r
s
m
BBIn+1 (s)
BBIn (s)
n
Nonlinear parameters of BBI
SD1
SD2
t
u

Figure S9 Ablation of PVT→CeA neurons abolishes anxiolytic and respiratory effects of preBötC^{Glu}→PVT stimulation.

a) Viral strategy combining photostimulation of the preBötC^{Glu}→PVT pathway with caspase-3-mediated ablation of PVT→CeA neurons. b) Immunohistochemical confirmation of ChR2-mCherry expression in the preBötC (left) and reporter expression (EYFP) in the CeA (right). Scale bars, 100 μm. c) The number of PVT^{Glu} neurons projecting to the CeA (EYFP^{+}) was significantly reduced in taCasp3-injected mice (right, n = 4) relative to controls (left, n = 5). d) Experimental timeline for functional phenotyping under unrestrained and ARS conditions. e) Percentage of time spent in quiescent or active behaviors during a 40-min recording (n = 7 per group). f) Cumulative time in active *vs.* quiescent states (n = 7 per group). g) Proportion of time allocated to each active behavior (n = 7 per group). h) Time-course traces of RF. Insert: Cumulative distribution of RF under unrestrained conditions (n = 7 per group). i) Ratio of LF to HF (LF/HF) (n = 7 per group). j-l) Quantitative analysis of CV (j), SDBB (k) and RMSSD (l) of BBI (n = 7 per group). m) Poincaré plot of BBI. n) Nonlinear parameters of BBI (n = 7 per group). o) Time-course traces of RF. Insert: Cumulative distribution plot of RF from ARS-treated mice (n = 7 per group). p) Ratio of LF to HF (LF/HF) post-ARS (n = 7 per group). q-s) Quantitative analysis of CV (q), SDBB (r) and RMSSD (s) of BBI (n = 7 per group). t) Poincaré plot of BBI post-ARS. u) Nonlinear parameters of BBI post-ARS (n = 7 per group). Statistical significance: All data are presented as the mean ± SEM. $^{*}p < 0.05$, $^{**}p < 0.01$, $^{***}p < 0.001$, $^{****}p < 0.0001$, as determined by two-tailed unpaired *t* test or two-tailed unpaired *t* test with Welch's correction (c, i-l, n, p-s, u), one-way ANOVA with Bonferroni's multiple comparisons tests (f, g) and two-way ANOVA with Bonferroni's, Tamhane's T2 or Dunn's multiple comparisons tests (h, o).

Figure S10

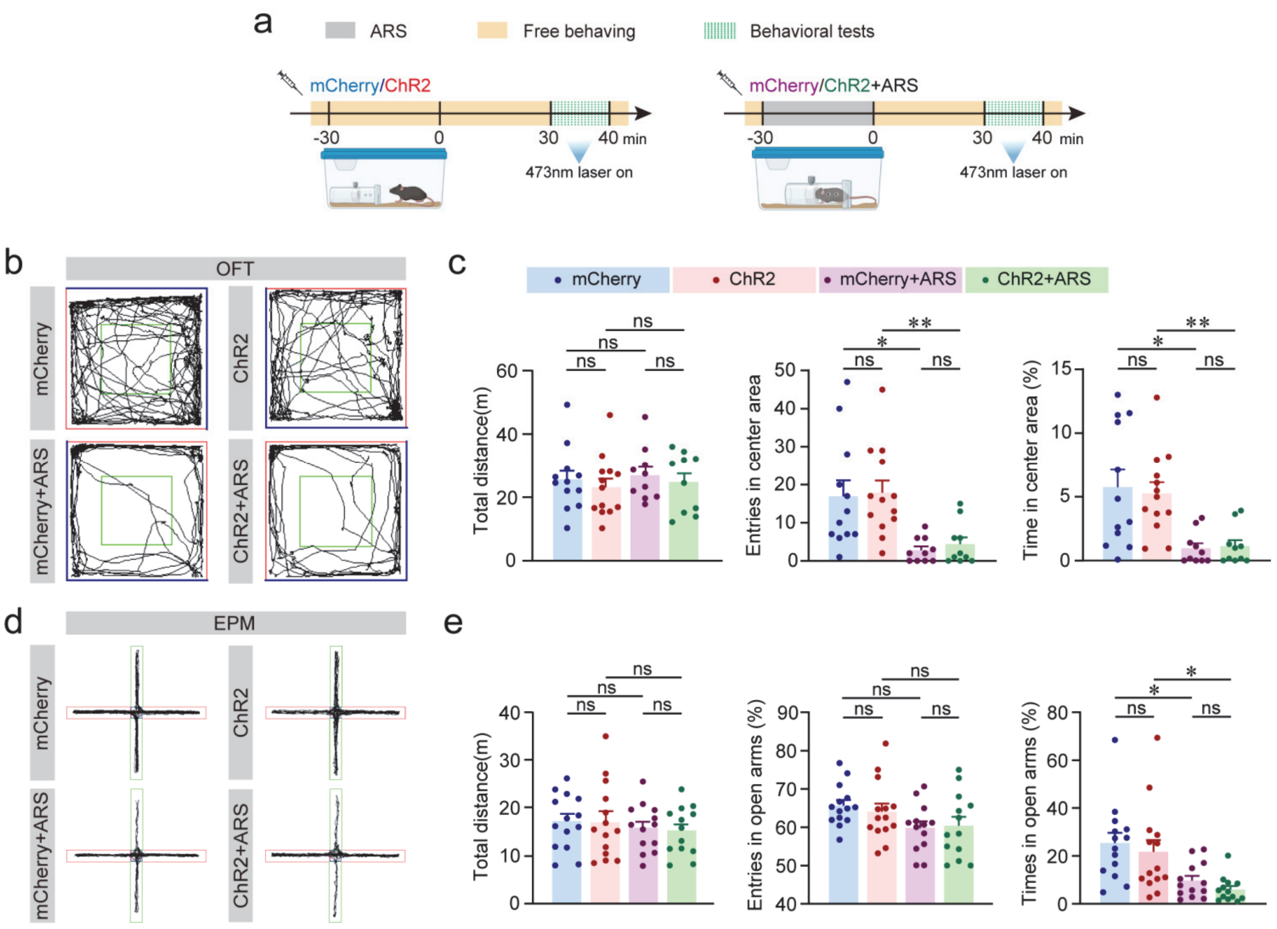

Figure S10 Ablation of PVT→CeA neurons abolishes anxiolytic effects of preBötC^{Glu}→PVT stimulation.

a) Timeline of combined optogenetic stimulation and ablation experiments. b) Schematic and representative trajectories of the OFT. c) Quantification of OFT parameters in unrestrained and ARS-treated mice before and after photostimulation (n = 12 for mCherry, n = 13 for ChR2, n = 10 for mCherry + ARS and ChR2 + ARS). d) Schematic and representative trajectories of the EPM. e) Quantification of EPM parameters (n = 14 for mCherry and ChR2, n = 13 for mCherry + ARS and ChR2 + ARS). Significance levels: $^{*}p < 0.05$, $^{**}p < 0.01$. Statistical analyses were performed using one-way ANOVA with Tukey's multiple comparisons test (c, e). All data are presented as the mean ± SEM.

Figure S11

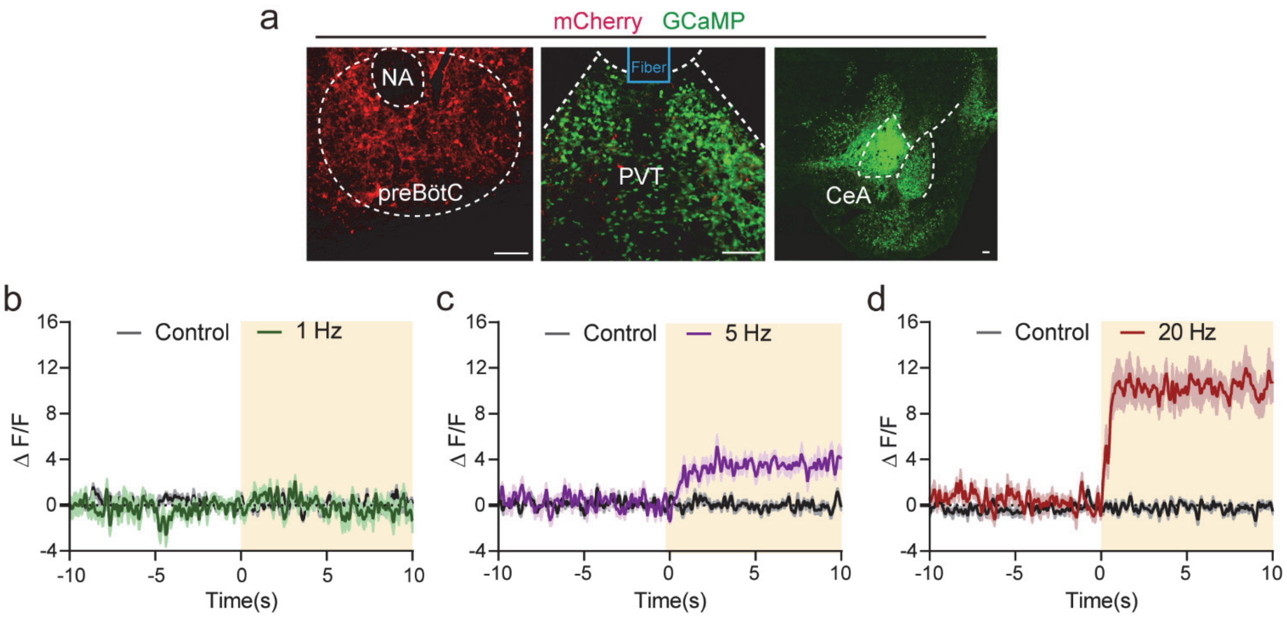

Figure S11 Photostimulation of preBötC^{Glu} neurons projecting to the PVT enhances activation levels of PVT neurons projecting to the CeA, related to Figure 6.

a) Representative images of mCherry-expressing preBötC neurons, GCaMP6f-expressing PVT neurons and GCaMP6f-expressing CeA neurons. Scale bars, 100 μm. b-d) Representative traces depicting changes in Ca^{2+} signals of PVT neurons projecting to the CeA upon photostimulation of preBötC^{Glu} neurons projecting to the PVT at frequencies of 1 (b), 5 (c), 20 Hz (d) (transition point at time 0 s).

Figure S12

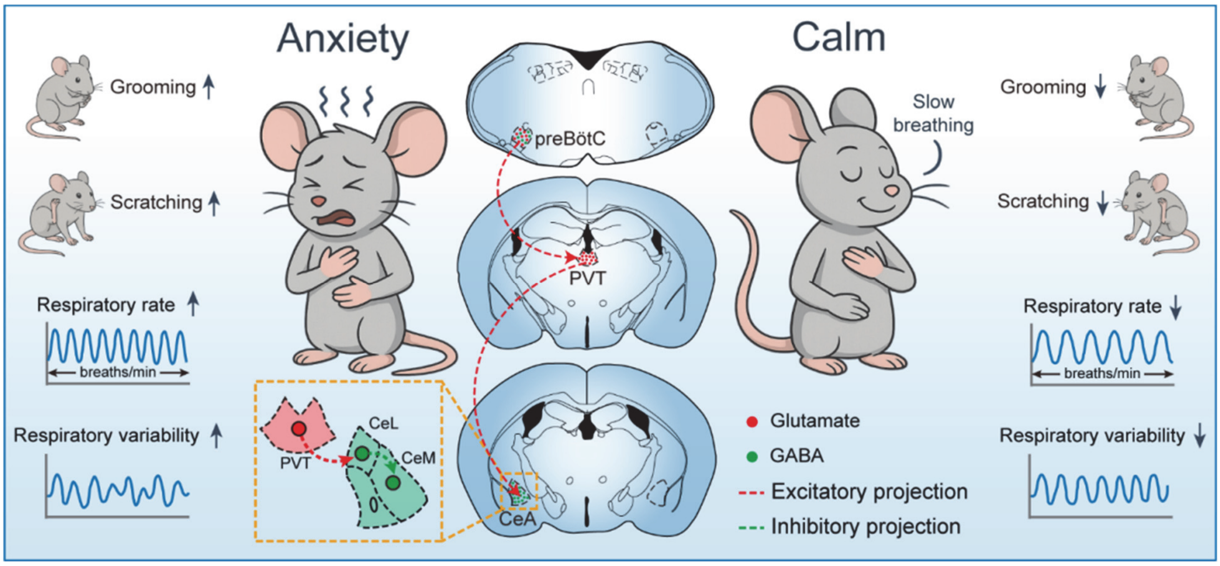

Figure S12 Establishing an ascending brainstem-thalamus-amygdala circuit for breathing-emotion integration.

We delineate a conserved preBötC^{Glu}→PVT→CeA circuit that bidirectionally gates anxiety and respiration. Functional interrogation demonstrates that activation of this pathway is anxiolytic and respiratory-stabilizing, while its inhibition has the opposite effect. Mechanistically, PVT exerts its anxiolytic action via a disinhibitory microcircuit: its inputs preferentially target CeL GABAergic neurons, which subsequently suppress CeM output. This model defines a dedicated ascending pathway for respiratory-limbic integration.

Table S1. The detailed statistical information for all figures of this study.
